# A Dual Origin of the *Xist* Gene from a Protein-Coding Gene and a Set of Transposable Elements

**DOI:** 10.1371/journal.pone.0002521

**Published:** 2008-06-25

**Authors:** Eugeny A. Elisaphenko, Nikolay N. Kolesnikov, Alexander I. Shevchenko, Igor B. Rogozin, Tatyana B. Nesterova, Neil Brockdorff, Suren M. Zakian

**Affiliations:** 1 Institute of Cytology and Genetics, Russian Academy of Sciences, Siberian Department, Novosibirsk, Russia; 2 National Center for Biotechnology Information, National Library of Medicine, National Institutes of Health, Bethesda, Maryland, United States of America; 3 Medical Research Council, Clinical Sciences Centre, Imperial College Faculty of Medicine, London, United Kingdom; 4 Department of Biochemistry, University of Oxford, Oxford, United Kingdom; University of Dayton, United States of America

## Abstract

X-chromosome inactivation, which occurs in female eutherian mammals is controlled by a complex X-linked locus termed the X-inactivation center (XIC). Previously it was proposed that genes of the XIC evolved, at least in part, as a result of pseudogenization of protein-coding genes. In this study we show that the key XIC gene *Xist*, which displays fragmentary homology to a protein-coding gene *Lnx3*, emerged *de novo* in early eutherians by integration of mobile elements which gave rise to simple tandem repeats. The *Xist* gene promoter region and four out of ten exons found in eutherians retain homology to exons of the *Lnx3* gene. The remaining six *Xist* exons including those with simple tandem repeats detectable in their structure have similarity to different transposable elements. Integration of mobile elements into *Xist* accompanies the overall evolution of the gene and presumably continues in contemporary eutherian species. Additionally we showed that the combination of remnants of protein-coding sequences and mobile elements is not unique to the *Xist* gene and is found in other XIC genes producing non-coding nuclear RNA.

## Introduction

The majority of genes on one of the two X chromosomes are inactivated in female mammals during early development. It is postulated that this phenomenon is required to equalize X chromosome gene expression in males (XY) and females (XX) [1]. In eutherians X inactivation is controlled by the X-linked inactivation center (XIC). The XIC is a complex genetic locus comprising of a number of genes producing non-coding nuclear RNAs [2]. Key amongst these is the X inactive specific transcript (*Xist*), that plays a pivotal role in X inactivation. *Xist* RNA spreads *in cis* along the X chromosome to be inactivated, initiates transcriptional silencing, and triggers chromatin modifications that maintain an inactive state of the genes [3].

Duret *et al.* [4] proposed that the XIC genes *Tsx* (*T*estis specific X-linked gene), *Xist*, *Enox(Jpx)* (Expressed neighbour of *Xist*), *Ftx* (Mus musculus *FTX* non-coding RNA), and *Cnbp2* (Cellular nucleic acid binding protein 2) originated through pseudogenization of the protein-coding genes *Fip1l2* (Polyadenylation factor I complex (Saccharomyces cerevisiae), like 2), *Lnx3* (Ligand of numb-protein X 3), *Rasl11c* (RAS-like, family 11, member C), *Uspl* (Ubiquitin specific peptidase), and *Wave4(Wasf3)* (Wiskott-Aldrich syndrome protein family member 3), respectively. This assumption is based on the fact that the genes in question are located within the regions of synteny in chicken (*Gallus gallus*) and human (*Homo sapiens*) and contain homologous regions. *Xist* may have evolved in part from the *Lnx3* gene, which in chicken produces an mRNA encoding a protein similar to PDZ domain containing ring finger 1 (LOC422320, ACC XM_420296.1). *Lnx3* exons 4 and 11 show similarity to exons h4/m4 and h5/m6 of human and mouse *XIST/Xist*, respectively. The function of *Lnx3* in chicken is unknown, and this gene ceased to exist in eutherians after contributing to *Xist* formation. Six exons of *Fip1l2* show similarity to the orthologous region of human and mouse. Three of them correspond to mouse *Tsx* or human pseudo-*TSX*. Four *Rasl11c* exons are homologous to sequences in the canine and bovine XIC, and one *Rasl11c* exon displays similarity to a sequence in the human XIC [4]. The contribution of *Uspl* and *Wave4(Wasf3)* to the formation of XIC genes remains to be clarified.

In this study we have conducted an independent analysis of the origin and evolution of the X inactivation center and the *Xist* gene in eutherians using a set of bioinformatic approaches. We have compiled a consensus *Xist* gene reconstructed from sequences that had undergone considerable divergence in each species. As a result, additional data have been obtained demonstrating how the XIC originated from a region containing protein-coding genes. In particular, we have demonstrated that the genes *Enox*(*Jpx*) and *Ftx* of the XIC contain exons homologous to those in cognate protein-coding genes *Uspl* and *Wave4(Wasf3)*, and we have discovered at least three additional exons of *Lnx3* in the *Xist* gene sequence. Moreover we have found that many *Xist* exons originated from mobile elements of various classes, which gave rise to simple tandem repeats detectable in the structure of the gene. Based on the analysis of the *Xist* gene consensus, we have reconstructed an ancestral structure of this gene, which may have existed 100 million years ago (Mya) in the eutherian ancestor, and traced its evolution to contemporary eutherian species.

## Results

### *Xist* consensus

**Figure 1:**
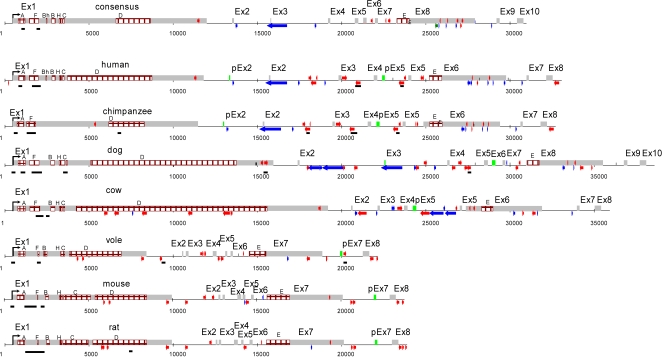
Organization of the *Xist* gene in seven eutherian species and the *Xist* gene consensus. Grey rectangles represent exons; green, pseudoexons (pEx); blue arrows, LINEs; and red, SINEs; brown rectangles indicate tandem repeats (for details see table 3); black rectangles, CpG-islands. Note that in chimpanzee *Xist* gene tandem repeats (B, Bh and C) are not detected possibly due to gaps in sequence. Pseudoexon (pEx) means a part of an intronic sequence that is present in other species as an exon.

To examine further the origin of the *Xist* gene we have generated an *Xist* gene consensus from genomic sequences of rat, bovine, canine, and human *Xist/XIST*, belonging to four eutherian orders (Rodentia, Cetartiodactyla, Carnivora, and Primates, respectively). Based on PipMaker [5] comparison we removed all species-specific repeats (e.g. species-specific SINEs, tandem repeats, pseudogenes etc) from the annotated *Xist* sequences and then aligned the sequences by ClustalX [6]. The resulting alignment was then manually corrected. The *Xist* consensus sequences were generated by the AnnHyb program (http://www.bioinformatics.org/annhyb). The resulting *Xist* gene consensus contains 10 exons and 500-bp flanking regions at the 5′ and 3′ ends. The size of the genomic locus is about 30 kb (Table 1, Fig. 1). The correspondence between exons in consensus, human and mouse *Xist* is shown in table 2. The consensus includes all known types of tandem repeats (A–F) (Table 3), which are essential components of the gene. Additionally we detected a previously unrecognized repeat H (Table 3) and mobile elements (Fig. S1) in the *Xist* consensus sequence, that were reconstructed from fragments found in the nucleotide sequences of the gene in different species. The *Xist* gene consensus sequence was used in further analyses described below.

**Table 1:** Relative length of *Xist* elements.

Species	Gene size	Length of homologous exons										Total intron length	Total exon length
		Ex1	pEx2	Ex2	Ex3	Ex4	pEx5	Ex5	Ex6	Ex7	Ex8		
C.f.	37592	15480	96	59	140	211	162	130	4561	195	370	16188	21404
B.t.	34934	18693	90	-	137	210	164	131	4524	156	372	10457	24477
H.s.	32063	11333	90	64	137	209	161	164	4543	146	378	14838	17225
P.t.	32050	11316	90	64	137	209	144	164	4541	146	378	14861	17189
		**Ex1**	**Ex2**		**Ex3**	**Ex4**	**Ex5**	**Ex6**	**Ex7**	**pEx7**	**Ex8**		
R.n.	22898	9430	83	-	137	213	141	154	4610	146	387	7597	15301
M.m.	22786	9483	91	-	132	211	147	155	4521	141	340	7565	15221
M.r.	21161	7939	84	-	138	213	103	134	4361	134	384	7671	13490
consensus	30297	11506	93	58	138	209	185	169	4678	151	374	12736	17561
**consensus**		**Ex1**	**Ex2**	**Ex3**	**Ex4**	**Ex5**	**Ex6**	**Ex7**	**Ex8**	**Ex9**	**Ex10**		

Notes. Pseudoexon (pEx) means a part of an intronic sequence which is present in other species as an exon. Abbreviations: exons (Ex) and pseudoexons (pEx), in *Canis familiaris* (C.f.), *Bos taurus* (B.t.), *Homo sapiens* (H.s.), *Pan troglodytes* (P.t.), *Rattus norvegicus* (R.n.), *Mus musculus* (M.m.), *Microtus rossiaemeridionalis* (M.r.), and consensus *Xist* gene; (–) the exon is missing. Length is shown in base pairs.

**Table 2:** Correspondences between consensus, mouse and human *Xist* elements and their putative origin.

*Xist* elements	Human *XIST*	Mouse *Xist*	Origin from *Lnx3*	Origin from transposable elements
Pmin	Pmin	Pmin	Exon 1, Exon 2	
c1	h1	m1	Exon 2	TR, TE
c2	ph2	m2		LINE, L3CR1
c3	h2	-		LINE, L1MC3
c4	h3	m3		LINE, L1
Intron 4	Intron 3	Intron 3	Exon 3	
c5	h4	m4	Exon 4	
c6	ph5	m5	Exon 5	
c7	h5	m6	Exon11	
c8	h6	m7		TR, TE
c9	h7	pm7		Fragment of ERV
c10	h8	m8		Fragment of DNA transposon

Notes. Abbreviations: exons of the mammalian consensus gene – (c1–c10), human and mouse exons, respectively – (h1/m1 – h8/m8); pseudoexons – (ph, pm). Minimal promoter – (Pmin). Tandem repeats – (TR), transposable elements – (TE), endogenous retrovirus – (ERV). Pseudoexon (pEx) means a part of an intronic sequence which is present in other species as an exon.

**Table 3:** Tandem repeats in the *Xist* gene.

No	Repeat name, exon	Description	References	Possible origin
1.	**A** exon 1	Repeat A is present in all the species studied and is the most important region of the RNA for chromosome silencing. It has a monomer with an average size of about 50 bp. The copy number varies from six to nine. This repeat consists of two parts—constant GC-rich and variable AT-rich.	[11, 17 26, 27, 28]	endogenous retroviruses
2.	**F** exon 1	Repeat F is not purely a tandem repeat and is detectable in all species only as a 19-bp motif. Its copy number varies from two to five. This region in mouse carries the P2 promoter.	[15]	DNA transposons
3.	**B^h^** exon 1	Repeat B^h^ is detectable only in primates; it is a small microsatellite tract, (C)_5–8_A	[15]	
4.	**B** exon 1	Repeat B is also a microsatellite tract, (CCCCAG)_n_; it is present in all the species.	[15], [26], [27], [28]	
5.	**H** exon 1	Repeat H is a small block of tandem repeats with a monomer unit of 33 bp, which is detected in the consensus gene and is most pronounced in dog. The monomers in contemporary species have diverged beyond recognition.		L1 LINE
6.	**C** exon 1	Repeat C is a block of tandem repeats with a monomer of 11 bp and copy number of 14 present only in mouse and rat. In other species including rather closely related voles, only one incomplete monomer is detected.	[15], [26], [27], [28]	endogenous retroviruses
7.	**D** exon 1	Repeat D is the largest and most obscure. Monomers have mutated considerably and lost their similarity during evolution; however, fragments of mutated monomers with various length reamplified in even-hoofed ungulates, carnivores, and primates. The monomer with a length of 290 bp amplified in primates. Bovine displays three different blocks of D repeats with the monomers of 93, 95, and 189 bp. Two similar 94-bp monomers amplified in dog. All the consensuses of the monomers in different species are relatively homologous; however, considerably distinct variants are also found. This repeat forms an essential part of exon 1 in human, chimpanzee, dog, and bovine.	[15], [26], [27], [28]	endogenous retroviruses
8.	**E** exon h6/m7	Repeat E is the most variable repeat present in all the species. Its monomer is 30–25 bp long, AT-rich, and not distinct.	[15], [26], [27], [28]	Intracisternal A particle (IAP)

### Homology of *Xist* and *Lnx3* exons

**Figure 2:**
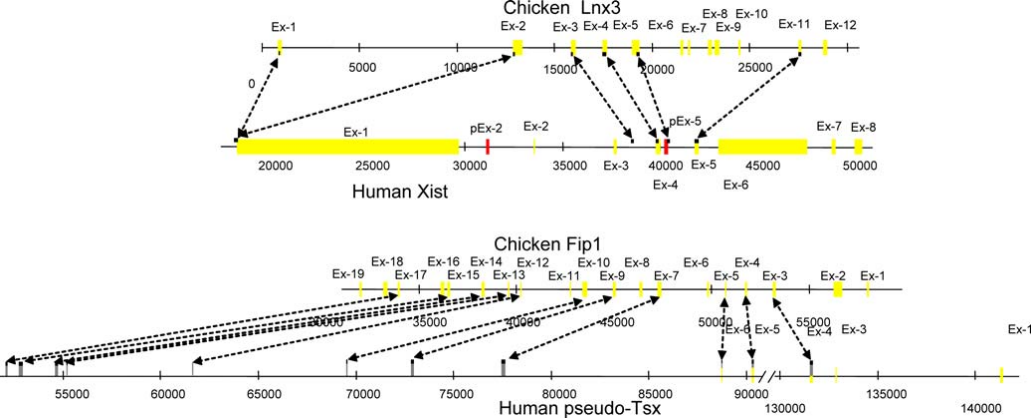
Comparison of the chicken *Lnx3 –Fip1l2* genomic region with the human *XIST*, 3′-flank and pseudo-*TSX* region. Homologous regions are indicated by arrows. Positions are shown in base pairs. Coloured boxes represent exons; yellow, pseudoexons; and blue, 5′-UTR.

It has been shown previously that chicken *Lnx3* exons 4 and 11 have similarity to human *XIST* exons 4 and 6 [4]. Using FASTA, SSEARCH and WUBLAST programs we performed a more comprehensive comparison between the chicken *Lnx3* gene and human *XIST* to obtain additional evidence that *Lnx3* is the precursor of *Xist*. In addition to the previously found similarity we discovered that *Lnx3* exons 3 and 5 are similar to human *XIST* introns 3 and 4 (59 and 60%, respectively). Interestingly, human *XIST* intron 4 corresponds to rodent *Xist* exon 5, which displays 65% homology to *Lnx3* exon 5 (Fig. 2, Fig. S2). We also discovered additional similarity between the *XIST* promoter region and *Lnx3* exons 1 and 2 (Fig. 2, Fig. S2). Moreover, the same fragment of ancient LINE, belonging to the L3 family (widespread in the genomes of the majority of vertebrate species), is located at the orthologous position in *Lnx3* and the *Xist* consensus 5′ region. The similarity between the chicken mobile element and mammalian L3 consensus is 64.7%. Comparison between the *Xist* consensus described above and chicken *Lnx3* revealed the same homologous regions as in human *XIST* (Fig. 2, Fig. S2). The P-value indicates that the similarities are statistically significant (Table 4). Therefore, our data substantiates and adds to previous observations that eutherian *Xist* consensus has extended homology to *Lnx3* in the promoter region, exons 1, 5–7, and intron 4.

**Table 4:** P-value for similarities found between *Xist* elements in different species (estimated by program PRSS from FASTA package ).

First sequence	Second sequence	PRSS P-value
*G.gallus Lnx3* exon3	*H.sapiens Xist* intron 3	2×10^−5^
*G.gallus Lnx3* exon5	*H.sapiens Xist* intron 3	0.01
*G.gallus Lnx3* exon5	*M.musculus Xist* gene exon 5	0.004
*G.gallus Lnx3* exons 1, 2	*H.sapiens*e *Xist* gene (ACC U80460) promoter	0.007
*G.gallus Rasl11C* ex3	*H.sapiens* Xic locus	10^−9^
*G.gallus Wasf3* ex1	*H.sapiens* Xic locus (*FTX*)	10^−5^
*G.gallus Wasf3* ex2	*H.sapiens* Xic locus (*FTX*)	3×10^−6^
*G.gallus Uspl* exon 1	*H.sapiens* Xic locus (*ENOX*)	0.006
*G.gallus Uspl* exon 2	*H.sapiens* Xic locus (*ENOX*)	7×10^−5^
*G.gallus Uspl* mRNA (ACC NM_001031123.1)	human *ENOX* EST BC071776	0.0007
Consensus *Xist* exon 2	L3 CR1	0.0001
Consensus *Xist* exon 4	L1 LINE	3×10^−5^
Consensus *Xist* exon 9	ERV2	4×10^−5^
Consensus *Xist* exon 10	DNA transposons hAT-10_XT	0.13
Consensus *Xist* exon 10	ZOMBI DNA	2×10^−5^
Monomer of consensus tandem repeat A	Endogenous Retrovirus ERVB5	2×10^−5^
Monomer of consensus tandem repeat B	versus Endogenous Retrovirus ERV18_MD_I ERV1	0.0006
3-mer of consensus tandem repeat F	DNA transposones HAT2_MD hAT	2×10^−7^
Monomermer of consensus tandem repeat H	L1 LINE	0.001
Monomer of consensus tandem repeat C	Endogenous Retrovirus ERVB4_3-I_MM	2×10^−5^

### Contribution of transposons to the formation of the *Xist* gene

We compared consensus exon sequences with the database of chordate mobile elements using the program WUBLAST 2.0 and elevated search sensitivity by setting the word size W = 3 instead of the default value W = 10. We identified a short fragment of MIR3 in *Xist* exon 1. The consensus exon 2 (m2/ph2) is similar to the ancient L3CR element (Fig. S3, Table 2). Unexpectedly, exon 3 of the consensus, which corresponds to human exon 2, has similarity to an L1 fragment and is present in the spliced transcript (ACC M97168). The boundaries of this human exon are inside the L1 sequence. i.e., human exon 2 is composed of a 64 nt fragment of a mobile element which is included in mature *XIST* RNA (Fig. 1). Presumably, a similar phenomenon takes place in the splicing of canine *Xist* RNA too (Fig. 1). Consensus exon 4 (m3/h3) is also similar to L1 sequence (Fig. S3). A considerable part of exon 8 (h6/m7) is similar to mobile elements of different classes (SINEs, LINEs, and DNA transposons) (Fig. 1). Exon 9 (pm7/h7) shows 64% similarity to endogenous retroviruses. Exon 10 (m8/h8) displays the highest similarity to DNA transposons. Almost the entire exon displays a 61% similarity to the hAT-10_XT element and the central part, 66% similarity to the ZOMBI transposon (Fig. S3). The degree of sequence identity of consensus exons 2, 4, 9, and 10 to mobile elements varies from 59 to 64%. All similarities found are statistically significant (Table 4). This is also the case for the degree of similarity between consensus exons 5, 6, and 7, and the exons of the *Lnx3* gene. Thus, we have shown that *Xist* consensus exons 2–4 and 8–10 share some homology with different classes of mobile elements (Fig. S3). Additionally, our data suggests that after the divergence of the main mammalian taxa, young species-specific repeats (Alu of primates, B1 and B2 of rodents, *etc.*) and pseudogenes were integrated in the *Xist* gene (Fig. 1). Some young taxon-specific SINEs were inserted in exon 1 and are now present in the processed *Xist* transcript. Interestingly, *Xist* CpG islands show a unique distribution in each species and are predominantly coincide with the insertion of species-specific mobile elements (Fig. 1).

### Tandem repeats in *Xist* originated from mobile elements

The majority of *Xist* exon 1 consensus consists of various tandem repeats (A–F), which may represent up to 78% of the exon, as observed in bovine *Xist* (Table S1). The beginning of exon h6/m7 also contains a tandem repeat E (Fig. 1, Table 3). Using the program TRF4, we deduced the consensus sequences for the main blocks of tandem repeats in the *Xist* gene and compared the consensus dimers with the database of dispersed repeats (RepBase). This analysis revealed that repeats A, C, and D display a similarity to different classes of endogenous retroviruses (Table 4, Fig. S4). Three similar variants of repeat F are detectable in the *Xist* gene consensus and they all display some similarity to DNA transposons. The ancient block of H repeats, which we identified in the *Xist* consensus sequence, displays a high similarity to the 3′ end of marsupial (M. *domestica*) LINE L1-7_MD L1. Repeat B is a microsatellite tract (CCCCAG)n, which is found in dispersed repeats and could have been introduced, for example, with the same endogenous retroviruses (Table 4, Fig. S4). It should be noted that a weak similarity to the endogenous retroviruses is found between blocks of tandem repeats. The comparative analyses allow us to conclude that tandem repeats, which are the main part of exon 1, could indeed have originated from fragments of endogenous retroviruses and other mobile elements (Table 4, Fig. S4).

### The ancestral *Xist* gene and its evolution

**Figure 3:**
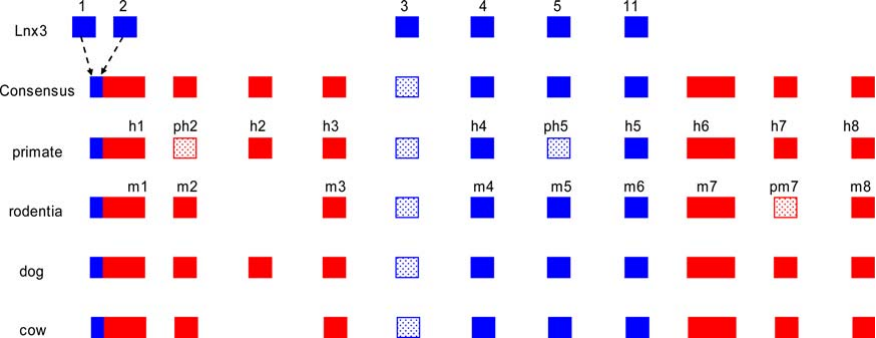
The *Xist* gene evolved from a protein-coding gene and a set of transposable elements. Blue box – exons originating from *Lnx3*; red box - exons originating from transposable elements; dashed box – pseudoexons.

By compiling the *Xist* sequences representing four eutherian orders, we generated the *Xist* gene consensus and recognized a number of ancient elements which diverged considerably in the contemporary eutherian species (Fig.S1). We suggest that the consensus in some extent reflects the *Xist* structure that existed approximately 100 Mya in a common ancestor of Eutheria. We propose that the ancestral *Xist* gene contained ten exons (Table 1, Fig. 1). Part of exon 1, intron 3 and exons 5 to 7 are all remnants of *Lnx3*, whereas exons 2 to 4 and 8 to 10 originated from mobile elements of various classes (Fig. 3, Table 2). The promoter region of the ancestral gene evolved from exons 1 and 2 of the *Lnx3* gene, flanked by an ancient L3 element at the 5′ region (Fig. 3). It is likely that basic arrays of tandem repeats A, B, D, F, and H, which originated from endogenous retroviruses, were already present within exon 1 of the ancestral *Xist* gene as well as MIR3. It seems likely that the ancestral *Xist* contained a single copy of the C repeat as it is present in a single copy in the majority of species analysed (vole, bovine, canine, human) and is tandemly repeated in mouse and rat only. The proportion of mobile elements in the ancestral *Xist* gene is relatively low, representing only 4.39% of the total gene length (Table S2). In the *Xist* gene of contemporary eutherian species the proportion of mobile elements is higher than in the consensus gene and varies from 7.9 to 15.9% (Table S2).

The promoter region of *Xist* is among the most conserved regions of the gene, showing a degree of homology between 82% and 98% for different species. Rodents are the exception, as the degree of homology for the promoter region is only 68% when compared with members of the other orders (Table S3). Probably, exon 3 of the *Xist* consensus is retained in dog, chimpanzee and human but was deleted together with surrounding sequences in other species studied. The scenario for consensus exon 3 is consistent with evolutionary tree data that suggests that dog and primates diverged from the common ancestor earlier than primates and rodents [7]. The sequences corresponding to consensus exon 2 and 6 are retained as *Xist* exons in as evolutionarily distant taxa as rodent and insectivore [16], but are part of intronic sequences in other species. Conversely, consensus exon 9 which is retained in the majority of species is an intron in rodents (Fig. 1, Fig. 3).

During evolution the length of *Xist* RNA in different species changed dramatically. This occurred mainly due to formation of new exon–intron boundaries (for example, exon 2 of *Xist* in rat, exon 7 of *Xist* in mouse and vole), and variation in length of exon 1 by differential amplification of tandem repeats (Tables 1, 3, S1) and insertions of species-specific SINEs (Fig. 1, Table S2). However, despite almost twofold differences in exon size in various species, the total GC content in exons is conserved, varying from 41.4 to 42.6% (Table 5). The GC content in introns is more variable ranging from 37.2–41.9%.

**Table 5:** GC-content (%) in various elements of bovine, human, mouse and vole *Xist* genes.

Spe-cies	5′-flank				Exons		Introns		3′-flank		Gene Xist	
			P_min_									
	size	G+C%	size	G+C%	size	G+C%	G+C%	size	G+C%	size	G+C%	size
B.t.	20000	41.3	109	53.2	24470	41,4	37.6	10464	40.0	16000	40.3	34934
H.s.	18355	42.7	101	56.4	16974	42.1	37.2	15089	39.4	9582	39.8	32063
M.m.	21461	42.1	104	54.8	15080	41.6	40.6	7706	45.3	15759	41.3	22786
M.r.	11778	43.2	105	53.3	13357	42.4	41.9	7805	44.7	11452	42.2	21162
R.n.	18165	41,5	101	54.0	15155	42.7	41,6	7743	44,3	14325	42,3	22898

Notes. Abbreviations: Ex, exon; In, intron; Pmin, minimal promoter; H.s., human; C.f., dog; M.r. vole *Microtus rossiaemeridionalis*; B.t., bovine; R.n., rat.

### The time of *Xist* gene emergence

To estimate the approximate time of *Xist* gene emergence, we aligned the nucleotide sequences of chicken and opossum *Lnx3* genes (*Gglnx3* and *Mdlnx3*) with human and bovine *Xist* genes (Fig. S5). This alignment was constructed with preservation of the *Lnx3* reading frame (all the deletions/insertions causing frameshift were discharged from the alignment). *Xist* is not a protein-coding gene; however, this alignment allows the number of potential synonymous and nonsynonymous substitutions to be assessed, as all the stop codons in the *Xist* gene were removed.

**Figure 4:**
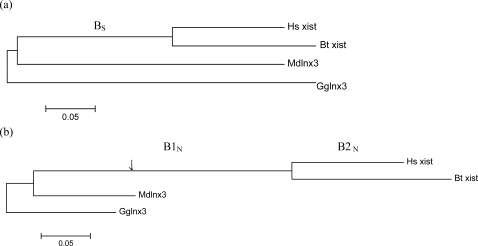
Tentative time frame of *Xist* origin. Minimum evolution trees constructed using (a) synonymous and (b) nonsynonymous substitutions (modified Nei-Gojobori method, p-distance) [24]. Arrow indicates an approximate time of *Xist* gene origin, assuming that this gene would remain a protein-coding gene and have a similar evolutionary rate as *Mdlnx3* gene. Scale is shown nucleotide differences per site.

Figure 4 shows the phylogenetic trees constructed for the synonymous and nonsynonymous substitutions. As anticipated, the evolutionary rates of synonymous and nonsynonymous substitutions at positions within the *Xist* gene are approximately equal. For the synonymous substitutions (Fig. 4a), the lengths of branches approximately correspond to the anticipated time estimates, thereby indicating the uniformity of evolutionary rates over time. Nonsynonymous substitutions (Fig. 4b) give characteristically short branches leading to *Gglnx3* and *Mdlnx3* (as compared to synonymous substitutions). This results from the action of stabilizing selection, which is typical for the majority of protein-coding genes. As *Xist* is not a protein-coding gene, this limitation is removed. Therefore, the branch leading to *Xist* genes is very long as compared with the other branches. If *Xist* were a protein-coding gene and displayed approximately the same evolutionary rate as *Mdlnx3*, the length of these branches would be approximately equal (the suggested position of *Xist* gene is denoted with arrow; Fig. 4b). However, the rest of the length of the branch leading to the last common ancestor of bovine and human (B1_N_ in Fig. 4b) approximately equals the lengths of the branches leading to human and bovine (B2_N_ in Fig. 4b). This indicates that the *Xist* gene has an ancient origin. The long branch B1_N_ might result from an acceleration in the evolution of the *Lnx3*/*Xist* gene during the pseudogenization process. The fact that B1_N_ is approximately 2 times longer (Table S4) in the Figure 4b (nonsynonymous sites) compared to B_S_ (Fig. 4a) can probably be attributed to the nonsynonymous sites being far from equilibrium with the mutational pattern at the moment of pseudogenization, such that for a while the number of substitutions per site would have been higher for these sites than for the synonymous positions (which would have been already closer to mutational equilibrium).

To test the reliability of the obtained results, we aligned nucleotide sequences of *Gglnx3* and *Mdlnx3* and all available mammalian *Xist* genes (Fig. S6). This alignment contained a much smaller number of sites (results not shown), however the results are similar to the results obtained for the four species alignment (Fig. S6). Similar estimates of branch lengths were obtained for different models of substitutions (Table S4) suggesting that the obtained results are reliable.

In general, a short alignment and a lack of good descriptors of the pseudogenization process prevent precise time estimates; however, taking into account that divergence of bovine and human is assessed as 90–110 Mya (branch B2_N_ in Fig. 4b) [8], we can assume that the time of *Xist* gene emergence might be approximately 2 times older than this estimate (in the assumption that the evolutionary rate of nonsynonymous substitutions at the branch B1_N_ is 2 times faster than B2_N_ and B_S_), i.e., 180–220 Mya. This estimate exceeds the assumed divergence time of marsupials and eutherians (130–180 Mya). Taking into account the inevitable errors in the estimation of evolutionary rates and branch lengths in short alignments, the data obtained suggest that the inactivation of the *Lnx3* gene occurred during the divergence of marsupials and eutherians or in early eutherians.

### The Origin of *Ftx* and *Enox*(*Jpx*) genes

Next we analysed the contribution of *Uspl* and *Wave4(Wasf3)* genes to the formation of the XIC, as this question has remained unresolved in previous studies. We conducted a comprehensive search for similarity between the chicken protein-coding genes *Wave4(Wasf3)* (ACC XM_420299.1), *Rasl11c* (ACC XM_420297.1), and *Uspl* (ACC NM_001031123.1) and the genes of the mammalian XIC. A more detailed analysis with less stringent parameters in the search programs detected several regions homologous to the chicken *Wave4(Wasf3)* gene in the human XIC. Exons 1 and 2 of *Wave4(Wasf3)* correspond to exons 2 and 3 of *FTX* (ACC AK057701.1), displaying 58 and 55% similarity, respectively. The similarity is statistically significant in both cases (Table 4). Note that the exon–intron boundary between two homologous exons of *Wave4(Wasf3)* and *FTX* coincides (Fig. S2). Moreover, the considerably diverged regions homologous to *Wave4(Wasf3)* exons 3 and 4 are detectable at the same human loci (localized to *Ftx* 5′ region). These data prompted us to search for homology to genes *Uspl* and *Rasl11c*. The considerably diverged remnants of the *Rasl11c* gene were found in a region between human *ENOX*(*JPX*) and *XIST* genes (Fig. S2). The most pronounced homology was for exon 3 of *Rasl11c* (63%), as described previously in [4]. The most likely explanation is that *Enox*(*Jpx*) is related to the chicken gene *Uspl*. As demonstrated previously, the main part of the *Enox*(*Jpx*) exons, except for exon 1, evolved from mobile elements of various classes [2]; moreover, different types of dispersed repeats gave rise to the exons of the gene in different mammalian species. When analyzing the human EST database, several alternatively spliced transcripts with common exons 1 and 2 were detected for the human *ENOX(JPG)* gene (data not shown). We found a statistically significant similarity (58%) between the putative *ENOX*(*JPX*) promoter region and exon 1 of chicken *Uspl*, between *ENOX*(*JPX*) exon 2 and *Uspl* exon 5, and between *Uspl* exon 7 and human EST BC071776, which is likely to be an alternatively spliced variant of the *ENOX*(*JPX*) gene (Table 4, Fig. S2). Thus, like *Xist*, *Enox(Jpx)* seems to have evolved from a protein-coding gene and a set of different transposable elements.

## Discussion

### The origin of the mammalian X-inactivation center

**Figure 5:**
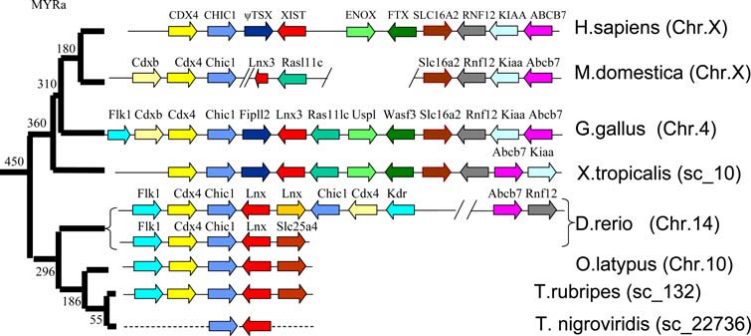
Comparison of the human XIC region with its orthologous sequences in other species. Comparison of the human genomic region containing protein-coding genes *CDX4*, *CHIC1*, *SLC16A2*, *RNF12*, *KIAA2022* (indicated as *KIAA22*) and *ABCB7* that surround the pseudo (ψ)*TSX*, *XIST*, *ENOX* and *FTX* genes with its orthologous region in opossum (*M. domestica*), chicken (*G.gallus*), frog (*X. tropicalis*) and four fish species (*Danio rerio*, *Oryzias latipes*, *Takifugu rubripes* and *Takifugu nigroviridis*) *(* *Table S5* *)*. Homologous genes are shown in the same colour. The orthologs of chicken *Rasl11c* and *Uspl* genes are detected on chromosome 18 of medaka fish (*O. latipes*) and on chromosome 7 of zebrafish (*D. rerio*). In fish, *Cdx4*, *Chic1* and *Lnx* are not linked with *Slc16a2–Rnf12* and *Slc25a4* flanks *Lnx* in *T.rubripes* and *O. latipe*. *Cdx4*, *Chic1* and *Lnx* genes are found at two different locations on *D. rerio* chromosome 14 and at one location *these* genes are involved in inversion duplication. Interestingly, two genes, *Tex11* (testis expressed gene 11) *and Dlg3* (discs, large homolog 3) that have orthologs on the human X chromosome in the region Xq13.1, are located downstream from *Kdr* (kinase insert domain receptor) in *Danio rerio (not shown)*.

In this study we analysed the origin and evolution of the group of linked genes from which the mammalian XIC originated. The tight linkage of *Lnx3* (the precursor of *Xist*) with protein-coding genes *Cdx4 and Chic1* (caudal type homeo box transcription factor 4; cysteine-rich hydrophobic domain 1), which flank *Xist* in eutherians, was detected in fish and, presumably, existed in the ancestor of vertebrates 450 Mya (Fig. 5). Unlike the evolutionary conserved syntenic group *Cdx4*, *Chic1* and *Lnx3*, the other genes that contributed to the sequence of the Xic, *Rasl11c*, *Uspl*, and *Wave4(Wasf3)*, are found within other linkage groups in fish (for details see Fig. 5 legends).

Tight linkage of all the precursor genes of the XIC and the flanking conserved protein-coding genes is also detectable in amphibians [4], [10], [11]. In pipid frog (*Xenopus tropicalis*), the genes *Cdx4*, *Chic1*, *Fip1l2*, *Lnx3*, *Rasl11c*, *Uspl*, *Wave4 (Wasf3)*, *Slc16a2* (solute carrier family 16), and *Rnf12* (ring finger protein 12) are detectable within the same scaffold (Fig. 5). Note that in an ancestral amphibian, the *Fip1l2* gene was inserted into the conserved syntenic group *Cdx4*–*Chic1*–*Lnx3*, detectable in fish (Fig. 5). In chicken the order and orientation of the genes remains unchanged and they are integrated with genes which represent the main part of X conserved regions in mammals. Thus, it is likely that the formation of a proto XIC commenced about 450–360 Mya in ancestral amphibians.

An independent disruption of this linkage group took place in Prototheria and Methateria [10], [11], [12]. It is interesting, that these genes map to different regions of an autosome, chromosome 6 [11] in monotremes, while in marsupials they map to the X chromosome [12]. This linkage group remained in the eutherian lineage, where the five protein-coding genes of the proto XIC underwent pseudogenization about 180 Mya to give different sequences of the X-inactivation center [4].

### The origin of the genes in the mammalian XIC

Our comprehensive analysis together with previously published data [4] has enabled us to outline the origin of genes in the X-inactivation center. *Cnbp2* is a retrotransposed protein-coding gene specific for eutherians [4]. Three exons of the protein-coding *Tsx* gene, which is expressed during spermatogenesis, originated from *Fip1l2*. The homology of *Fip1l2* exon 17 is found in the region located 2 kb from the end of *Xist* exon 8. We have also found additional exons of this gene in the human sequence (Fig. 2). Two genes, *Uspl* and *Wave4(Wasf3)*, provided the basis for development of *Enox*(*Jpx*) and *Ftx*, respectively. Both *Enox*(*Jpx*) and *Ftx*, contain exons homologous to those in cognate protein-coding genes *Uspl* and *Wave4(Wasf3)*, respectively. Additionally *Uspl* contributed one exon to the promoter region of *Enox*(*Jpx*). Presumably, *Rasl11c* was omitted from the evolutionary process and its remains are located between *Xist* and *Enox* (*Jpx*). Analysis of *Enox*(*Jpx*) and *Ftx* ESTs has revealed overlapping variants of their RNAs (BC345566 (*ENOX*), BM546361 and CN412172 (*FTX*)) similar to *Xist* and *Tsix* RNAs. It cannot be excluded that this is another pair of regulatory genes, however, their function and possible role in X inactivation is as yet untested. As for the origin of the *Xist* gene itself, the following scenario of ancestral gene formation seems the most probable. The basis was the *Lnx3* gene, containing Pdz and RING finger domains. *Lnx3* exons 4, 5, and 11 gave rise to the exons 5, 6 and 7 of the *Xist* consensus gene (Fig. 2). The *Xist* promoter region originated from the 5′-UTR of *Lnx3* exons 1 and 2. The remaining six *Xist* exons including those with simple tandem repeats detectable in their structure have similarity to different transposable elements and thus the *Xist* gene has originated from pseudogene *Lnx3* and a set of various transposons. These mobile elements were integrated into the locus and subsequently their fragments were incorporated into the RNA.

Simple tandem repeats A, B, C, D, E, and F contributed essentially to the formation of exons in the ancestral gene; their monomer units presumably originated from endogenous retroviruses as weak similarity was observed in blocks of tandem repeats (Fig. 1). There are some cases of coding sequences entirely or partially derived from ancient transposable elements [13], and it has been reported that over 4.4% of Unigene transcripts contain significant matches to transposable elements within their coding regions [14]. *Xist* can be classified as an intermediate pseudogene, and represents a new type of ncRNA gene. Our data adds to what is already known regarding *Xist* evolution by showing that insertion of transposable elements within the ancestral protein-coding gene contributed to the creation of a novel ncRNA gene. In fact, other regions of the XIC have evolved similarly by transposable element insertion, mutating active protein-coding genes into non-coding pseudogenes. Our results constitute important findings into the origin of the unique *Xist* gene and suggest that acquisition of transposable elements by a protein-coding gene may be a more wide-spread phenomenon contributing to ncRNA gene origin and evolution than previously thought.

### Species-specific mode of *Xist* gene evolution

Comparative analysis across different species shows that eutherian *Xist* evolved in a species-specific manner. As reported previously the unique sequence of the *Xist* gene is not conserved and evolves very rapidly [2], [15]. The exon-intron structure of *Xist* is also not strongly conserved. The interspecific differences in the unique sequence, length and structure of exons suggest that the length of the RNA (and, consequently, the sequences, like mobile elements and tandem repeats, contributing to the RNA size in different species) is either non-essential for function (neutral sequences) or these sequences became selectively adapted in a specific manner to the conditions of functioning in the genome and the X chromosome of particular species. Certain core sequences common for all species are essential for the regulation of gene activity and its function.

The functional role of introns in the structure of the *XIST*/*Xist* gene is not well understood. The introns in protein-coding genes can separate structural or functional protein domains; however, it is unknown what their roles is in regulatory genes producing nuclear RNAs and how they differ from exons, as neither are protein-coding. Moreover, there are some cases in the evolution of the *Xist* gene when exons convert into introns and vice versa. We named the exons that are inactivated in some species but preserved in introns as pseudoexons (pEx). Three such pseudoexons, which are active in some species and inactivated in others, have been identified (Table 1, fig. 1).

Alongside the formation of pseudoexons, which arise due to mutation to intron-exon sites (ph2, man; pEx2 - chimpanzee), we observed exons shortening at their 3′ ends due to the formation of new 3′–5′ exon/intron junctions, for example m7 (mouse) and vole exon 7 [15]. A similar phenomenon was reported previously for *Xist* exon 4 in mole, dog and cow [16].

Thus, the exon-intron structure of the *XIST*/*Xist* gene is not stringently conserved or evolutionarily stable. It displays lability and exon–intron transitions, presumably connected with species-specific patterns related to its function.

A characteristic of the *Xist* gene is the presence of both ancient and young species-specific mobile elements in the processed transcripts. A similar feature is observed in other XIC genes, *Enox*(*Jpx*), which also produce nuclear RNA. The processed transcript of the *Enox*(*Jpx*) gene includes SINEs, LINEs, and even processed pseudogenes [9]. Presumably, the integration of mobile elements into exons accompanies the overall evolution of these genes and continues in contemporary Eutheria. Possibly, this is a general mechanism involved in the formation and rearrangement of genes encoding large nuclear regulatory RNAs.

Despite the drastic evolutionary rearrangements of the *Xist* gene structure in different species, the 42% GC content in exons is a conserved characteristic of this gene in mammals and, presumably, is maintained by selection. The introns contain less GC pairs compared to the exons and display a considerable variation between orders. The total GC content in *Xist* gene is similar to the overall XIC region and varies in the range of 39.8–42.2% (Tables 4). The region of the minimal promoter differs drastically—it is enriched with GC nucleotides (52.4–56.4%). The 3′-flanking region of rodents is richer in its GC content compared with both the gene itself and its 5′-flanking region. In contrast, the 3′-flanking region in human and bovine displays a lower GC content compared with the gene; in addition, a decrease in the GC content is evident from the 5′- to the 3;-flanking region (Tables 4). On the other hand, the localization and structure of *Xist* CpG islands in the species compared are different. The detected CpG islands in human and bovine are mainly associated with SINE and LINE repeats. The absence of conservation in the localization of CpG islands and their divergence in the case of similar localization in the *Xist* gene of different species may indicate that species-specific mechanisms are involved in the regulation of its activity.

Thus, we have proposed a mechanism whereby the *Xist* gene may have originated. We suggest that the *Xist* gene lost the function of the protein-coding gene *Lnx3* and no longer contained any extended ORFs. However, due to transposon insertions and their partial subsequent amplification, new functional domains formed (for example, repeat A in exon 1 [17]). These domains then became necessary for the transcriptional silencing of X chromosome genes. We suggest that this example of how a protein-coding gene loses its protein-coding function by mutation and then gains a new function due to transposon integration is not an exceptional case, but is a more wide-spread phenomenon applying to other non-coding RNA genes and pseudogenes with new functions.

## Materials and Methods

The following sequences extracted from the corresponding databases of sequenced genomes at the UCSC Genome Bioinformatics Site (http://genome.ucsc.edu/) were used: human Mar. 2006 (hg18) assembly range = chrX:72449111-74160153; chimpanzee Mar 2006 (panTro2) assembly range = chrX:75160560-75392648; dog May 2005 (canFam2) assembly canFam1_dna range = chrX:60100000-60735000; mouse Feb 2006 (mm8) assembly range = chrX: 99509137-100404904; rat June 2003 (rn3) assembly range = chrX:91358074-91899712; chicken Feb. 2004 (galGal2) assembly range = chr4:11820349-12295219; opossum assembly MonDom 4.0, January 2006 from Ensembl (http://www.ensembl.org/index.html); vole *Microtus rossiaemeridionalis* ACC AJ310130; and bovine ACC AJ421481.

Computer analysis was conducted using modern versions of the following software packages: BLAST ( [18], http://www.ncbi.nlm.nih.gov/) for searching for homologous sequences; Tandem Repeat Finder 4 [19] for searching for tandem repeats; RepeatMasker [20] for searching for mobile elements; Fasta [21], CLUSTALX [6] for aligning two and more sequences (the programs and data are available at http://genome.ucsc.edu/, http://www.ensembl.org/, and http://bio.cse.psu.edu/) and (PipMaker, [5]) for genomic analysis of extended loci.

Comparative analysis of the *Xist* gene of ten eutherian species belonging to four orders (Rodentia, Cetartiodactyla, Carnivora, and Primates) was conducted. The structure of the *Xist* gene in four closely related vole species was determined earlier [15]. As vole *Xist* genes are very similar, the sequence of *M. rossiaemeridionalis* was used in the present study. We identified chimpanzee, dog, and rat orthologs of the *Xist* gene in the data available from genomic sequencing projects of the three species by pairwise alignments with the known human, mouse, and bovine sequences [2]. The exon–intron structures of dog, chimpanzee, and bovine *Xist* genes were determined by comparing with the human and mouse genes. Comparison with genomic sequences demonstrated that the exon–intron boundaries in bovine and chimpanzee genes coincide with numerous spliced ESTs. In dog only one EST is located in the region of *Xist* gene. Comparison with the genomic sequence demonstrated that it contained exons 2, 4, 5, and beginning of exon 6 (according to human *XIST* gene). This data cannot be regarded as final, because other species display various rare variants of EST splicing, including the loss of certain exons. The *Xist* sequences of different mammalian species were analyzed for the presence of both tandem and dispersed repeats using the TRF, RepeatMasker and the repeat database RepBase12.03. (http://www.girinst.org/repbase/index.html) [22]. To search for the homology between considerably divergent and evolutionarily distant sequences (for example, human–chicken), we used the programs WUBLAST with the parameter W = 3 and FASTA34 with the algorithm Smith–Waterman or SSEARCH34 and the parameters f = −14, g = −2. The similarity between *Xist* exons and mobile elements was searched for using the same programs and parameters. Each individual exon was compared with the database of chordate mobile elements, which was obtained by consolidating the human, primate, Mammalia, Rodentia, and Vertebrata databases (http://www.girinst.org/repbase/index.html). CpG islands of at least 200 bp and with a GC percentage exceeding 50% and with an observed/expected CpG ratio that is greater than 60 bp were detected using the FIND-CPG program from the PipTools [23]. To test the statistical significance of the obtained alignments the standard PRSS program from the FASTA software package was used (Table 4). As E-value and z-score estimated by the WUBLAST, FASTA and SSEARCH programs depend on sequence length and the size of search database, we assessed the significance of alignments by P-value calculated in the PRSS program from FASTA packed and selected the most significant alignments.

The modified Nei-Gojobori method as implemented in the MEGA program [24] and a maximum likelihood method as implemented in the CODEML program [25] were used to estimate the numbers of synonymous and nonsynonymous substitutions per site. Phylogenetic trees based on multiple alignments of nucleotide sequences were constructed using the minimum evolution method as implemented in the MEGA program with default parameters.

## Supporting Information

Figure S1 Nucleotide sequences of eutherian consensus gene Xist presented in GENE BANK format.
(0.10 MB DOC)

Figure S2 Alignment of the chicken proto XIC region with the human and mouse XICs. (a) Sequence alignment of part of human Xist intron 3 and a part of Lnx3 exon 3 (ACC XM_420296.1). (b) Alignment of part of human Xist intron 4 and part of Lnx3 exon 5. (c) Alignment of part of mouse Xist exon 5 and chicken Lnx3 exon 5. (d) Alignment of part of the human Xist promoter and Lnx3 exons 1 and 2. (e) Alignment of chicken Rasl11c exon 3 and the 5′-region of Xist. (f) Alignment of chicken Wasf3 exon 1, 2 and the 5′-region of Xist. (g) Alignment of chicken Uspl exon 1, 2 and the 5′-region of Xist, (h) Alignment of chicken Uspl mRNA (ACC NM_001031123.1) and human ENOX EST BC071776. (a–g) computed with SSEARCH34 from FASTA package; (h) computed with WUBLAST 2.0.
(0.05 MB DOC)

Figure S3 Homology of the mammalian consensus Xist gene exons 2, 4, 9, 10 and various transposable elements.
(0.03 MB DOC)

Figure S4 Homology of consensus monomer sequences of the main blocks of tandem repeats in Xist gene and various transposable elements
(0.03 MB DOC)

Figure S5 Nucleotide sequences alignment of cDNA of chicken and opossum Lnx3 genes (Mlnx3 and Clnx3) and human and bovine Xist genes.
(0.04 MB DOC)

Figure S6 Minimum evolution trees constructed using (a) synonymous and (b) nonsynonymous substitutions (modified Nei-Gojobori method, p-distance) [18]. The numbers for the interior branches refer to the bootstrap values with 1,000 pseudoreplicates.
(0.03 MB DOC)

Table S1 (0.04 MB DOC)

Table S2 (0.04 MB DOC)

Table S3 (0.03 MB DOC)

Table S4 (0.03 MB DOC)

Table S5 (0.06 MB DOC)
