# Supplementary material for: A Dual Origin of the Xist Gene from a Protein-Coding Gene and a Set of Transposable Elements
Source: PLoS One. 2008 Jun 25;3(6):e2521. doi: 10.1371/journal.pone.0002521 (PMC2430539; doi:10.1371/journal.pone.0002521)
Supplement: Figure S1 — Nucleotide sequences of eutherian consensus gene Xist presented in GENE BANK format. (0.10 MB DOC) [file pone.0002521.s001.doc]

lOCUS conxist 30986 bp DNA linear ROD 17-SEP-2007

DEFINITION Consensus xist 4 species human, rat,cow, dog.

ACCESSION

VERSION

KEYWORDS .

SOURCE Canis familiaris, Bos taurus, Homo sapiens, Rattus norvegicus

ORGANISM Eukaryota; Metazoa; Chordata; Craniata; Vertebrata; Euteleostomi;

Mammalia; Eutheria.

REFERENCE 1 (bases 1 to 30986)

AUTHORS Evgeny A. Elisafenko, Nikolay N. Kolesnikov, Aleksander I. Shevchenko, Igor B.

Rogozin, Tatyana B. Nesterova, Neil Brockdorff, Suren M. Zakian

TITLE A dual origin of the Xist gene from a protein-coding gene and a set of various

transposable elements

JOURNAL Unpublished

REFERENCE 2 (bases 1 to 30986)

AUTHORS Shevchenko,A.I., Elisaphenko,E.A. and Zakian,S.M.

TITLE Direct Submission

JOURNAL Submitted (17-SEP-2007) Developmental Epigenetics, Institute of

Cytology & Genetics, Aca. Lavrentieva Ave 10, Novosibirsk 630090,

Russia

FEATURES Location/Qualifiers

source 1..30986

/organism="Mus musculus"

/mol_type="genomic DNA"

gene 508..30804

/gene="Xist"

exon 508..12013

/gene="Xist"

/number=1

repeat_unit 810..1253

/gene="Xist"

/rpt_type=tandem repeat A

repeat_unit 1485..2075

/gene="Xist"

/rpt_type=tandem repeat F

repeat_unit 2492..2524

/gene="Xist"

/rpt_type=tandem repeat Bh

repeat_unit 2804..3052

/gene="Xist"

/rpt_type=tandem repeat H

repeat_unit 3257..3443

/gene="Xist"

/rpt_type=tandem repeat B

repeat_unit 3481..3589

/gene="Xist"

/rpt_type=tandem repeat C

repeat_unit 6654..8709

/gene="Xist"

/rpt_type=tandem repeat D

exon 13512..13604

/gene="Xist"

/number=2

exon 15780..15837

/gene="Xist"

/number=3

exon 19215..19352

/gene="Xist"

/number=4

exon 20813..21021

/gene="Xist"

/number=5

exon 21300..21484

/gene="Xist"

/number=6

exon 21996..22164

/gene="Xist"

/number=7

exon 23265..27942

/gene="Xist"

/number=8

repeat_unit 23323..24064

/gene="Xist"

/rpt_type=tandem repeat E

exon 29220..29370

/gene="Xist"

/number=9

exon 30431..30804

/gene="Xist"

/number=10

repeat_region 3303..3442

/rpt_family="(CCCCAG)n"

repeat_region 11148..11172

/rpt_family="AT_rich"

repeat_region 11441..11576

/rpt_family="MIR3"

repeat_region complement(13735..13830)

/rpt_family="L2c"

repeat_region complement(15575..16774)

/rpt_family="L1MC3"

repeat_region complement(18430..18510)

/rpt_family="HAL1"

repeat_region 21769..21875

/rpt_family="MIR3"

repeat_region 22636..22677

/rpt_family="MIR3"

repeat_region 22786..22822

/rpt_family="MIR3"

repeat_region 22847..22922

/rpt_family="MIRb"

repeat_region 23066..23137

/rpt_family="(TTA)n"

repeat_region 23951..23991

/rpt_family="(TG)n"

repeat_region complement(25567..25742)

/rpt_family="MER58C"

repeat_region complement(25778..25828)

/rpt_family="MIRm"

repeat_region 25858..25900

/rpt_family="MIR3"

repeat_region complement(26212..26254)

/rpt_family="L2"

repeat_region 26306..26355

/rpt_family="MIR3"

repeat_region complement(26791..26836)

/rpt_family="L2"

repeat_region 26866..26926

/rpt_family="MIR_Mars"

repeat_region complement(27089..27143)

/rpt_family="L2B_ME"

repeat_region 27156..27225

/rpt_family="MIR3"

repeat_region complement(28913..28957)

/rpt_family="L2"

BASE COUNT 8255 a 5834 c 5163 g 9480 t 2254 others

ORIGIN

1 cccttgaaga gggttctaac cttaatgtat tacyccttcc tagcttcttt ggttcmwawg

61 tytggtggag gagttataca ttctggctga atgaggtatt tggatacttt atcaacatat

121 caaagctctc cccctccttc ctcccattty caaaaccaaa ctaaactaaa accttttcat

181 ttgttatgct gtttgtggag aattttagca ttagttatct aatttgattt actcaacgtc

241 cctattttat agataagaac ccattgaaaa cccattgaag ttgtgmctcc tggtctagaa

301 atgaagscta cttccagtmt aatgttcttt ctagtatgtt gctgttttct tgaaatmtcc

361 ctttttcttg ttactgggta aattttgaac cagctaaacc acaaagatgt ctggctttca

421 atcttctagg ccactcctct tctactccct ccgccctcag tycccccccc ctcacttctt

481 aaagcgctgc aatttgctgc tgcagccata tttcttcctc tcccgagamt ggaagcttmc

541 taacagtgga tctctccgcc cgtggggttc tttctggaac attttccagm ccccaaccac

601 cccttatggc gtatttcttt aaaaaaatac accaaaattc cataaaatat ttttataatt

661 ctaaactttc tcctagtgtt ttcttgacac ctcctccata tttttcagat atttggaata

721 tttttaggca attttccatt ttaaaggaat ttttctttgg aatgattttt ggttgacacc

781 tctgtttttt ggggtttcct tttctttttc accctctttt ctatattwtt tgcccatcgg

841 ggctgcggat acctggtttr ymtattatta tttttattat tgcccaacgg ggccgtggat

901 acctgccttt taattctttt tttttaaatt tgcccatcgg ggccacggat acctgctttt

961 aatttttttt ttycccctta gcccatcggg gcctcggata cctgctgtgt acccccccyw

1021 mwraacsccc aacaccctgg cccatcgggg caacggatac ctgcttttaa aaaattttct

1081 tttcctggcc catcggggcc tcggatacct gctttaattt tttttwtttt cccttgccca

1141 tcggggcctc ggatacctgc tttaattttt kttttcctgg cccatcgggg ccgcggatac

1201 ctgcttagat tttttttttt caccgcccat cggggctttt tatggatgga aaartgttgg

1261 tttttgtggg tcgttgaact atctggaata tctacaaatt tttttgctgc taatcgtttg

1321 gtgttgtgtg agtggaccta cggctttggc agagagatga ctttgcagtt aagctaaggg

1381 ggtggtcagg ctggggagga aagatggccg ccactttaga sttgccgcmc aactcggctg

1441 agagctagty gtttgtgcta agtaaaacta ggaagccaag atggataacg ggacaggcag

1501 aggaaatcat atgcattgca tgagctaaat gtmtttgaat gagctgattt ggagcttgtt

1561 aggagctttg catgaattgt ggtatcatga ggtagaaaaa carrgratca tcttgtgtca

1621 tgttacaagg grctagtsaa aaatgaaagg gagaagactt aggcacaggg ttcaaaatgg

1681 cgattttgac cttgcggcat tgctcagcat ggctctctgc tttgttagag tgtccaaaat

1741 ggcggatcca rttttgccgc agtgttcaag tggcgggaag gccacatcat ggtggtgtct

1801 ttgttctagt gtgcagcatg gcggtagaaa tattcggtca cacaggaaaa gatggcggct

1861 gaagtacttg ccgcagtgta aaacatggcg ggcttttgtc tttgccgtgt gcatttcctg

1921 acaggttttg ccgcagggac aatatggcag accttgtcat ttggatatca tggcagtttg

1981 tcacgtggac atcatggcgg ggrtstttgm ccgktacatt cttggcgggc tttgcaccag

2041 gtgggcctgc cgcattgtta aagatggcgg gctttgccgc gtktgccacg caaagcgaaa

2101 gaaaaggtgg gatggacaat gttggattgc cgcatgaccc aaccaatcag aaagggtggt

2161 agaattgggc acagccaatt agtgsagagg atggaattag actgacwcac tgtccmrywa

2221 skmsgtgagt tagcatagca cctcacagcc gtctttattc agccagtcag gagammgaag

2281 tggaggggcc acgtttgtac ttctcccagt gggcggtacr msaggkgtty ccaaggtctt

2341 tccaaggaca tttggccttt ccacytyctc cctcccctct mattggctcc ctcccctcca

2401 gcattgcctc ttgcagtgct ggatatttgg ctgtgcaggc tgaacctcac cccattcctc

2461 tgcattggtg ccgtggccta aggctagcct cccccmcwyc cccacccccc gccccctact

2521 cttcctgcac tgtggccagg ggcagtgctc catgcctgct aggtgtgaac atggtggtga

2581 gccgtggcaa gaaccagaat ggatcacaga tgatcgttgg ccaacaggtg gcagaagagg

2641 aatccctgcc ttcctcaaga ggaacaccta ccccatggct aatgctgggg tcggattttg

2701 atttatattt attttttgga tgtcagtcat acatrgtctg attatgtggt ttgctagtgy

2761 ttggatttaa gccttaagtg actattatag aaatgtattt aggagcttta tttgtagaat

2821 tcattttaat tacatttaat gagttttcat ttttgggttc cttaaaattc cttaaagttt

2881 tcagtttctt ctttataaat tscttaamct ttttttggca gtagataktc aaagtcaaat

2941 wwctaacatt taaaaatttt rctrtttttt tactttgaaa ttgacctaat aaactacttc

3001 cctttgaatt tttggagtct tataacacag aaataattaa aaatttaact ttatgaccga

3061 atgtaaaaag gaatatttga aggtccacac aagtccaggc cttgctttgt tcccatcctt

3121 aatgctgcac taattgacta atgacctatc aatcwgcagg aaacttgaat tgctgtggtc

3181 ttgtgtcctc tattcaaact tattattgat tggaawwtta mtgcccayaa tttcaatttt

3241 ccgtggtmcc ctgcctccac agcacccagt tcctccccag ccctgctccc agcaamcccc

3301 tgccccagcc ccagccccag tcccagtccc aaccccagcc ccagccccgg cccagcccca

3361 gcccagccca acccctgccc yrmycysccc cagccctagc cccagcccca cmcccagctc

3421 cyyccagmcc cagccccagc ccasttctcc ctaaagtcat tccaattttc attgattcag

3481 tgctaaaaat aagttgtcca ttgctcatcc tataagactg agataccctg tctacctcta

3541 gcattgctga tcttcagtac tgactaccta agtcaccatt ttcagttaat gcacacaatc

3601 ccatttgtcc atgatcccat gttagaacaa agaatccatg taccctttac gttaatgtta

3661 agaaacgtca attramtgag agctttatat cagacaatta ttgcccataa ttttagttac

3721 tcatcatctc ctcaatcccc tgcccttaag ggagaaacca ttcctctgtc attgctgcag

3781 tagtcacagt cccaattttc tgagcagtgm tcttktctta tmtactgtgc tgaccaccta

3841 aamyctttgc attgagtgaa attctaattg cccataatcc tacccattgg attagacccc

3901 agtccmaaac ccacttgcat tcagcaaagg gtgcagaaaa cacaagtctt ttyggacagt

3961 taamatgctc agtcccaatt gtcatagctt tgtccattaa acaaaggcac cctacmamgt

4021 ttgccctgct tctggggart cctgttcttg gacaattaaa gtaccaaatt gtaattgywm

4081 attgtcttmc ccattaatca atgaccctca gtcscctttg cattgctmtt aggcagtgct

4141 gactacctga gaccatgttc cttgaattat taatgagtar aatcccaatt rtccatakyy

4201 ctgttmatta cacaagtcct ttgcarmytc tttgcattat agcagaggtg ctcatcctma

4261 aagtccctta ctttggaata ttaatggata caatmgttat tgttcatggt cctgcstaac

4321 agataagacc cactcatgct cttttcattg ctcctaggta gtgcggacta cctaccacct

4381 tgcattaata tgtaaaatmc tcatttccca tggtcccacc mattagtcta ggatatmyct

4441 tatctctatg ccattgcttc tgagttctga ttacccaagg tcccttctct taaacagttg

4501 atgtgcataa ttgcatatat ccatggtsct gtgcaataaa aaatggaatc ccaccycaty

4561 cyamcctctg tgggatattg ctagggagtg cagattattc aaraactcag ctcttggaca

4621 gttaatttgc acagttgcaa ttgtccagag tcctgtccat ttgaaagggc cactgtatcc

4681 tatttgcatg ctasaacgtg ggctgatcac ccaaggrctc tcctcttgaa ttgataamrt

4741 tcataattgc atttgtccac gatcttgtaa actagccaca cccactccct ttgtattcca

4801 gcaggggacc cttactactc aagacctctg cactaggaca gtttatgtac acaatcctaa

4861 ttgattagaa ctgagtcttt tgtatcaaga accctgcacc atctytgctt tacatctaaa

4921 agggtgctaa ttacctaagg cccctmtcca aaaattrtts atgtgcaaaa tgyratytcc

4981 ctatctgctg ttagtctagg atctcattcc mcccaaactc cctttgymtt actgcagagg

5041 gtacttggga ctgttaatgy gcwtaawtsc arytrtrgtc ttttcyatta aactaagatc

5101 ccawmysctc acaccctctt agcattacag taaagggtgc taaacactaa ggccactcct

5161 tttgtgcctg ttaatgtgca tagttgcatt tgtccctctt cctctgcact ggataaagac

5221 cccactcatt tcccttgtat tcagcagtgg atgmccttta stcaagacct ttgtactagg

5281 ataaagtgaa ccatggcaat tgaccacrac tatgtctttc asttcagttc cttgtattct

5341 gttttayarm aakrkatatt aattacctcy rttacttttc tctgggacta tttatgtgca

5401 aaattccart gttyawggyc cttctcttta aattaatatc ccaccctttt tacattatat

5461 caggggatgc tggtaaccca aactccttct cttgggactc ttaatgtgca taattatyyr

5521 tkmctyttwm tstgcatasa staaattccc tkayawtymr caatggattt cmtttattta

5581 agacctttgt aytmkgwtaa aatgtatrkt aattgactac actgmgtctt ttattsaagt

5641 ttttryatkm aaamttctag tgtttaaary ccttctcttc aaattaatat cccrcccttt

5701 ttgcattata tcaggggatg ctggtrtccc aaagtcyttt tcttgggatt ctwaatgtgc

5761 atrtgccctc mtgataagct aatatcttta attcttagta ctatamcaga gastactgac

5821 trccwgragt ttttctgttg gacagttaat gtgcacaatt gcactagaat cccatccccm

5881 tcacaccttc ttmgcatcag gggtgctaac caactagctg cttcttctcy tatacagttt

5941 aaatgtgcat aattacagta gtccatgctc ccttatgtta gamtaggatc ccmtmtcctt

6001 gccccattta cattactgca ggggcttctg actagccaag attcactctc ttggactgtt

6061 aatgtgcata cttacatttg ctgctgtacc tgtgcaccag gtaaggaccc tacccattct

6121 atttacattc cagcaggagg tacctactac tcaagatcct atacactaat acagttaatg

6181 tgcacaatct tagttgtctt gtacacatts tcagttgtcc acagctgtgc cttttagatc

6241 aggactcctg cacttatcaa agcagagggt actmaccaat ataaagccct tctcatggga

6301 ctgtagatgt atgtaattgc aattgtcaat ggtccttcaa gttaacttgg gtccctgacc

6361 cttcacatcc tctttgcttt actgcatggg gtactgtcca cttaaggccc ctttctcaaa

6421 ctgttaatgt gcataatgac aattacatta ggatccttct kacactccct ttgaagtaca

6481 acagggttgm tgacccataa ggtcccattt cttggcctgc caatawtgca tgattgtatt

6541 tgtctggatt tctgtgcact agagaaggaa akyactcccc tcctcactmc ctttcccttc

6601 cagcagggag tgcccactcc ataagaccct tacatttgaa cagtcaaggt gcacaactgt

6661 aastgaccac aaccatgcac cttggacatt aatgtgcgta attgcacacg gctcattcca

6721 tktgaataag gtcctactct cagacccctt ttgmaataca gaagggrtgc tgatcactaa

6781 ggcccctttt crtggcttgt rattatgmct gattgtarts gtccrtgrtc ctgtrcacta

6841 maraaggama sccctctccm cattcmtsws tykyrccttt ccctttcagc agggagtgcc

6901 cattctmtaa gacccttata tttgracagy maaggtgcat aattgtmatg aacacaacca

6961 tgmmccttgm acattaatgt gcataactgc acmcagctca tcctatctgg atataatcct

7021 actctcarac ycmykttgca gtacagcagg ggtgctgayc wmyaaggycc cctttcstgg

7081 cctgstaatg tgmmtgattg tatttgtccs ggtttctgtg tactagasar ggaaacctcc

7141 ccccacccct awtsttcctt tcccttccag cagggagtgc ccactgcmta agacmcttac

7201 gtttggacag tmaargtgca magttgtaak tgrccacaag catacrcctt gcayattaat

7261 gtgcatramc acyykgccca ttccatttgg atawgrtcct actctcamac cscttttgca

7321 gtacagcagg ggwtrctgat cmmyaaggcc ccwtttcttg gcctgttakg tgcrtgattr

7381 tatttgtctg grttcctgtg tactagasaa ggamacctcc ccccactccc tttctttaat

7441 ttccagcagg gagtgscccc tctataaggm crttacattt gmacartcaa ggrgcacaat

7501 trtaastgac cacagcsatg caccttggac attaatgtgc mtaaytgcac atggcycmty

7561 ccatcgaata aggtmctact stcagayscc ytttgmagta cagcaggggt actgatcact

7621 aaggcccttt ttcrtggctt gttattatgm ctgattgtat ttgtccmwgt ttctgtrtac

7681 tamasaagga aaccmctctc sccaytymwr tcttcctttc cctttcagca gggagtgccc

7741 attmcmtrag acccttatat ttgracarym aaggtgcama attgtaaktg accatamgca

7801 trmmccttgm acattaatgt gcataactgc acmtggctca tccmatctgr atawgrtcct

7861 actctcarac ctttttgcag tacagcaggg gtgctgaaca ctaaggcccc tcmmtyttcc

7921 tggsctktta tgtgtgtaat tgtatttgtt ccrgttcctm tgcamtagat arggarrcmt

7981 ccccmcaccc ccacttcttt cccttccagt aggrggtgcc crctmcwtaa gacctttaca

8041 tttggacagt saargtgcac aatttgtatr ttmccacaac cmtgtccctt gsakrwytca

8101 taaatmtrtg taytmctgca twmywttttc tcysrragtw yytwcatgca maattccary

8161 tgtmtaaggt cctacartct cacaccccct ttgcagtaca gcagrgggtg ttgatcaaca

8221 aagcccctct tcstggcmtg ttaatatgtg tgattgtatt tgtccstgtt tygtgcacta

8281 aagaaggaar ycwcccctgc cmttaaatct tcctttccct ttcagcaggg agtgcccact

8341 mcmtaagacc cttacatttg aacagttaat gtgcaaaakt actctaagaa yscmtymtgc

8401 cagcamccat gcaccaggrg wysywatywa cykrmtgcys yttctkttgg acattaatgt

8461 gcataactgc acacggccca tcctmtctwa tggaatagga tcctacactc aaaccccttt

8521 tgcagtacag caggggcrtg ttaaccamta aggtccactt tcctggactg ttaatatatg

8581 taattatatt tgtccmsgta tctgtgcact agamaaggaa gycccctctg cccctacrtm

8641 mtctgccttt mcatttcagc agggaatgct cactmttcaa gaccctaaca cttggacagt

8701 taatgtgcac aactatcagt gaccacaasc atgmrycttg garacttatg tgcattaact

8761 gcacacggct yatsctrttt gaataagrtc ctactctcaa ccccctttgc agtatagcaa

8821 gggtrctkay cactaaggcc cctstctttg ggctgtsast rkgygwttgt ctatgttcct

8881 gtgtactaga tttaggagaa ccttctcatr ractcccttt gctttactac aaggagtacc

8941 gaccacttta agatccttat atttgtacaa agtacatggt tttaattgac cacaccatgt

9001 cccttgaaca ttaatgtaca taattgcaac atagttcatc ctaatwaaac acagttgtac

9061 cttmtyamcy tycatttgca mtataccagg gttgctgmcc ccccaagkyy ccttttcttg

9121 gcctattaat atgcataatt gmatttrtct tggttcttgt gcactagaca aggatgcryc

9181 cccwcacycc ctacgccttc cartagttgg tgcccactgc taatgacctt tacatttgga

9241 cagttaatgt gcagaattgc agttgtccac aaccctatca cttccaggac cattatacct

9301 cttttgcact actgcagggg atactatttc cccccaaggt cccttctagt ggactattaa

9361 catwcataat tgaaattttc ttttgtcttt gtcagtagac targgtmata ccccctcacc

9421 tatcctttgc agtacatcag agtgtactga tcaaccaagg tcctctttgg actgttaata

9481 tgtgcaatta catttmctcc tgttctgtgc actagataag gatcccacct actcycttag

9541 catsttcagc aggtagtgcc cactactcaa gacttgtcac ttggaatgtt catgtgcaca

9601 akyarttstc taagcacgtc ttatgykaga acycctgtac cmcctttgct ttagagcagg

9661 gagatgctat tcactaaatg ccccttctct tggacttcaa tgtgcataaa tgcaattgtc

9721 catctcttct tttggactag gattgcacct cctcatactc cttttacacc aggggcatgt

9781 taattactaa tgaacccttc tcttgataat gttaatatmt ccccgymttt cttaytgtgg

9841 acctgtgtag tagaaaagga tcctatgtcc ctaccastgc cctttggatt gctgctgagg

9901 agtgctatca taacctcagc tcttggacaa ttaatatgca ccaataacat atcaarsgya

9961 tgatcwttag ataggacccc tgtaccctcm ttgcatacat caaggctact aaccyaaggc

10021 cccttytttt gkactgttag tgtgaatatt tgcaattacc tatgtcccct tctattagag

10081 ttaggacatt attccctmat accccctttg cattactaca ggggctgctg actacacaaa

10141 acttcccctg ggactgttaa taggcacaat ggcagttagc aatgggtttt tcctccctga

10201 ccttgttaag caaacacccc catctacart ttcccatggc ataataaagt ataaacattg

10261 caatatgccg taaacttgtc tatcaaacag tgacccatat tcccactcct tttgcattgc

10321 tccagtgtat aaaattccaa atagtcatgg cctcaccctt tatatwatct tccctctccc

10381 rgccccttgc ttgcccttgg acccttctta tggactattg ctcacaatct caggtgtcca

10441 tctttmcagc yatkagataa gattgtgccc cctcccscst cccaccactc ccctgcccct

10501 tttgcatctt tgctgggmaa tgttgactga gcaaagccct ttctcttgaa cttaaaaygt

10561 taacamtccc agatgtcatt gstttaccca ctttatattg ctgtaatgcc tcagttgcac

10621 tttccttggt cccacccaty akacatggac ccctccactt ccttttgcat tacttctgag

10681 tagtgctgac tacccaaagc cccttctgtg ttattaacac agtactgatt gtcacatttt

10741 tcagcccatc agcccaagat ctccctacca ctttgatgtt atttgtgcag tattgactac

10801 caaaagcagg ccagaactag gtggatgagc cttcactcct tttcctgcat ttgttaatga

10861 tcccaattcc aattattgtc acattctggg gacaggaacc attcctgccc acctctgtta

10921 ctgctttact gtgcaaaata ctcaaggcaa rgtcagaccc agggagctgg attgccaccc

10981 tttatttggg gtttccatta taagtatyca cttgaaaatt gtctccccaa gaaggaaggt

11041 tagcactttc tctgcattct tccttccaga gcagattgcc tggctaagaa tctcttttck

11101 yssctcttgt atattgctat tgtacagtgc caattgccag gatacaacca aaaaatttat

11161 ttatttatta atatttttat tttttaagaa agacatctgg attgcaaggt ggaattgata

11221 acctggtcat taaatttttg aagtcaaaaa acccatttat accatgtacc tgatgaccag

11281 tgtctctcat tttactgagg gtggtgagtc tgtggataga mcactgrmyy ttgatatttt

11341 aatatrccaa agatgttcta gagtagaact cttaagacca gtatctttgg gctctaccac

11401 catttwmaaa ycaytmyttg ggctttacca gcattcactt ttagaaaaac tacctaaact

11461 ttataatcct taaatttctt catctggagc accaacywsy ccctacttat ttcaagaaga

11521 ttgctgtaaa agaattaaat gagagaacat atgctgaggc gcttttgaaa accataggcc

11581 accttgwtta atttattact awataaaatg taccctattt aattctrgta ctaatttaaa

11641 gtagcttgaa gcaccacact gaagtgagga cttagaaatg atgggaccag tttccccatt

11701 ttatattaaa agaaaaataa gccaagatct aatcattctt ttggatataa atttcaacag

11761 tgagatagct gccgaatama aatgaataat atcccagcct ctagtgtaca gggtgtttyg

11821 tggcacagaa gtatctaata tggaactgct gaagcaaata actagtcatc acaacagcag

11881 ttctttgtaa tcactgaaaa agaatactat ttctctgaga aggatgtcaa aagatccgcc

11941 cagctcaggg tgcagtttgc actactagct ccttggacag ctgtaagaag agtctctggc

12001 tctttagaat actgtaagta ctactttgta gctattaagt aatcttttcc ctattctatt

12061 ttctttctct tagatgccac ccacagaaaa gtcagagggt ccwgtaagtt tccttccatg

12121 ttcctacctc atctgcaatr tatatataaw tayatryatr yawaaataym cccatatgag

12181 ttaacaassr aaactrtaga attaatattg tgtacccagc cctatgctag gttacactga

12241 aatgaggggt agaaatgatg tmatcctata taatctcatt cctgagatga ttatattgtt

12301 aaagagctaa tataagcaca cttgaaataa ctttagaaaa taataatcaa gtactgtttt

12361 gtgtgaatta taagttatag agttttaaaa agggagccca caagggtttg aagttgatag

12421 gataaactta aagaggtggg cttacttttc ctctttmatc caggamasga ttamtattgc

12481 agccatctgg gtagtccagt tggtttattt taatgtcatt tgtttttaac ctcttttgaa

12541 ggcatggaaa gagataagaa ataaagcctg gccattgtgt ttggctatat gccaaggctg

12601 gcaaattgtt gattgctaaa tctcataagc ttgagttttt caaagttcag ggattgggag

12661 tataaatctg atatgttggc tctttctaca attttactmc attcctgcca aagaacagat

12721 gammgtagta atgcctrttt agtctgagat attttttwag tttggaacct gtttggcaag

12781 agggcaatag agaaatgwaa gttttataaa accatgacct tccctacsta cyttmtgctg

12841 kagttwatat accctttcct ctcacctttk tcaayrrata cyttggtaag ctaaaaagmt

12901 tagcctccct gatgtaaaaw rrrrtccagc tgaaaaaata tyktttaaca taaaacttaa

12961 aacacttttt ttggtgcaca tccatgcatc acagcaagag caasaagaac catataaatg

13021 agctggcttc tcttactgcc cattttaatt catatccata tttcctcaca gcttgtttcc

13081 ccccagtgcc taaagacatt tgtctgactg actggaggta atatacaaat ggtcttgagc

13141 cttggaaaca tggtctrtgc cattgtttct caaactgaag taatgcgtca ggatgaaaca

13201 ccttctaaag gaacaaaatt ttctgagatc ctaaaaaata aatgttttga ggaacactga

13261 cttaasaaag ttatttgaaa tgtaaacatg ttttccaatt tcaagrtatc tttgtcaaag

13321 atatayaata ttttatataa tttatataga acttggggat ccatgagaat atatccacaa

13381 acccccaggg ttcttatcac cccaatttga gaaacactgg tctatgctta tgagatcttt

13441 tattgttast aatcaaattr tcattcgmtg ttaatataca actataacta ttattggaat

13501 ttgttttaaa gggatgaatt cggagctggt tctgtacccc cactcaagag gaaggatgga

13561 tcaattttag gtggagtgaa gcctgcacta gacagcatcc aaaggtgaat cttggagttg

13621 tatttcaatt cattgcttga aataaagtct ctagcamttg taraaaggga tgctctggaa

13681 atgtgggcat cttcaaaatm gagataatcc ttgtgttary tcaacaaayw ttattggacc

13741 agamactgga ataaatagca aaaccaaaga caggattcct gtcaaggaat ytgctwtcty

13801 atggwggatg cagaagtgaa caratcatta tggtgttggg carraayrcy tagwmtttrr

13861 tyctgrctyy rwsyttgrtc arwtcaccat maayytsasy rtctmgrgay kgctartgaa

13921 wtaagayagr grcttrawta yctmratccc tgaaaatgay attttgtsat ttggaaaatt

13981 tkmarrmrty taatyttttt tggcatttct ytaartgrtt wttrtswttt cwtttctgac

14041 tttyyycwtt ataraacctt aayatryagg attggaggaa gttttctgac cmttttcyca

14101 tatccycytt yagytttatc tttctgtaac ttmcatttct ctagccacct ccctaaattr

14161 yagaagaywr traraccyag ggytgctrtg rytrrrcatt cataatttct tttcaggrtr

14221 tytgkgccct grttakcaar tgtayasctk samrggagtt ywtgwcttaa gtaawgaatt

14281 aaragttgay ctgctaaaat attcttatwt gtgaaagyrt cctggraraa trykttrcca

14341 gcttaaagag aarraaactt atayctsaac tgagtstkct sttccccaaw ycttatmwgt

14401 ttggatkgca aagasttgat gtgtyrgttr aacagagcta atgccttcyk cyccwwgtct

14461 tamaraytgg mttggrwgaa aattgatwtw ytcamtacca tattttgrgc tstagrcaar

14521 tagcayttwa cacwggtktc cytaraaatc maactcaagt tggakctcaw gtatttraga

14581 cwtagctgry cyrctgaatt taacaagtya aacttcartg gccatgyaym gttatatatc

14641 aytrtatwta tggcttygas ttggytgytt ttrttggtga yttaggcttt acttsatwgc

14701 tcttccttga cctttccaaa ttgaryactg akayrtggar yttrggctyc ttstryayyy

14761 trtacaaatg agtttgrtaa agaactytcy tttactrttg atkyttatat trgaaataac

14821 ttttgattty ttcatgttag grtgagaray tgaarcaaaa tktaaatttk accrgtgcta

14881 gayttcttar atkatgggta gacttaaara ttattttytt raccaaytwg aatgctattc

14941 tagtktyccc rgaaayawka gargytatgc agyagaccca agyrataccy tyttattrca

15001 taatyrartg cktrtaamtt tmaaaatagg gatatgrctg grasatcast gtaywttacy

15061 rggtyyyatt ataaartrtc tatrttacyt wayccatarc tytgaaaact wktrgcatas

15121 tatattttay wgtatkytgt tagtrtgmtt gscattkway agtgwtggka trywrtyact

15181 cayaatctat atrtyattar agttttccag ccytayakrt ctccyttgac tgaaaaytrg

15241 ctataamtya ckrcytmykt tttayaryrg attgastagg tcyttccakr wrrtckgtkg

15301 atgwacarra ayaaagttta attgctaatk traataactt tttgatatta ygratwcytr

15361 rtatttgggc ctyatrttty aacwtyaaaa ykrmctaywa twaawyctmt aaacagaaaa

15421 gaaarakyaa gtctttagrt cagatytgcm aacaatgatg rtacstactg tagmaraatc

15481 tggaamayag amttaccagt tctyagrttc yattttgctt gctttwwaaa aamgyrycty

15541 ataagtcttc agmawctrtt ggsagattta raaraaawaa cyttytwatk ttagagrytt

15601 acagaatgrt tytgaagata gagttyctgt rtacyycrca cccaktttwy cyyaktgtta

15661 acattttaya ktastwtgrt acatttgtca caayraacma atattgatac attrttrtta

15721 actaragtcc ataytywaya tttyctyagt ttttcyytaa tgytcttttt rtgttccagg

15781 atcmcatysa arataccacs ytgcatytar tagtcatrty tccttaggct cctcttrgtw

15841 atgacagttt ctcagactcy ttrttyttra tgrmmttsac mrttttgagg actaatggtc

15901 martattctr tagaatgtcy ctmtattgga atttgtctga tgttcttctc atgaytagay

15961 tgggyttgag tktttrggar saagaccaya rmrgtasast gccattcttc ttwtcwmrag

16021 tacatactat caayrtgacw tatcactgtt katgttrwcy wtwatcayyt gkctraggta

16081 ctrtttgyca rgtttctcca syrwamawwt arwmttwwtt tytccwttyc cmtactrtay

16141 tkkawrggaa gycactatgt acttaargaa tgggaarttr ysttccacct cwttgakggy

16201 kagkrtytac ataarttatt tggaattctt ytgcacagga tgycttttct ccwyaatkta

16261 tgtryttatt yaktcattta takcartatg rwctcakgra tattttatay tytrgrtwat

16321 aatwcaktat tacttwwttt tgttcarakt gttcyagctt tggccwttgg gagktctttc

16381 akttgrcttk rayayaaccc catyytkwtt gagcaytttc ttaytttykg aaytacaasa

16441 tgstyyagrc tcatttgyat rtctcytryc ysrrtttctk caaggagycy tgatwctttt

16501 tattggagar yartattaga aatcaagawg tgartgytak gtgygctcat trctmctggg

16561 tgtcatksct tcaaracctt ytsagttgay ragasyragg aratatayay wtgsatwyta

16621 ackyatatgc ayrtakytat waatatwtat atmtgtatyy atwttaarmt aaatgkgttt

16681 atacmkwygt ytccaactct aaycattgcc mcatgratyr ttatagtctc myctcyttgc

16741 ttatctgtta cytcccayty cyagmarcct ggcttkgttg ggarattttt ytgttmatrt

16801 asggtagtga gwryttgaca kttgcytcta tggktwrrtt tagggagakt ttagctrtag

16861 rktrytcytg aaactaraaa tgacysttyt rysctaamtg kttstscyag ttttgaaacr

16921 taraataggt tscagaaaca aacytttctt aaraacyagr atytayyyca awycacattt

16981 tgmcmytsat tttcagawta aattrytytg ataymsyyag gtaakctgtt ccttggktat

17041 gcatttmtty tytcmgtttt ttctrakagy taragkaccy tgagaacact gaggtgggar

17101 ggargrgaaa gryrtgttcw yaygtgrgrt aggaaarrty catttastga sctccagcta

17161 syyttccaaa gtrcywatwt aagayycaag gagtagatkt mttycttggc aaytgtaayy

17221 caaatryaat tttyaacmkt tyaattttak tcaagmwart kggtrtgctg wtywwaaagt

17281 gyyctgatta atgtcayrkg yattgcatat wrakyagyar yaaaataaya traawytrtr

17341 ttgrgyataa ttttaatatt tatrtyarat atttgagaca stgtttctsa artctgtata

17401 ayaagtttga yagtagggag stttyctyyc aaraaaaraw ttrttyagtg wrcaccwaca

17461 trwtcacysc ttagatycta crattamtat tttgctrtat ttgattaaac ktttaaawra

17521 aaratwttat ttcwyrtgct ktkttraygc mcttmaccaa aytrcytttt yaarctgctk

17581 taaryyctkk trtasyrcat ascaatgcta twtyytttca twtggcacaa aacmcattta

17641 tatattgttt kcytctsttc ttttctktaa kcyccaggca acaaaastag wacrytkgcc

17701 acyaatctgg camygwrrty ctwywttwtr awgtagtcat mtagmtgatc tamactrtyt

17761 tayrgtgaaa trwrartatk gtraaarttt trtagaaasc tccyatayyc ctgaswakcy

17821 wtgcmcakay cycasrgkya aaagacctga aytgwgrgam raymtgggtt targtaycac

17881 ytrsttacyt kytrtwtrwr taacattgak gkartytcat cttcwgrgty cccagtttcy

17941 ttagagaatg aaatgtykra ttayrtgaty wtwwtwyyyt tttkagtayr tgatyataat

18001 aycmyktyta gctgtgayrr ttctgtsyrr tkytggagta yttgaaycag mkggcyrgct

18061 rtgccactyr gttwttmwst cyawwrkayk ttgayatttt gttsrkctry ragatgaskg

18121 attcycaaaa ttcttgtcar tgaatawkga acyctaatry atggttctgt atcmgttcca

18181 aatgtaacca ttttytctrg ccttagattc cckytwaggg aaarggaatc tyyttgarta

18241 tktcatcacy atartaacaa aactagargg ctttgatcta aagcaasata ctcyrtaaat

18301 atgsttmaga agayytgrgg agactggaat agtyrttycc ttttagrtry cwgtgtataa

18361 atgaatttgw rctwsratcc rttwatttaa aawttctttw ggtryatttr cttgyayatr

18421 garygymcat ttacymtcat taatggrgtt tyaggaarsa gyagastaaa trcataaaya

18481 tgtatgaacy gccatgttta actggaagcc ygmatttggr artmaagtat ctaatyttar

18541 aytmratwag gatgggaags atgttgscaa gagattttga agcyyrttsw gctyatattg

18601 agaacatcat agaacagttt ggcstywytr wakctagaga rtagwgttga ataagtgats

18661 ttycayatay tcytgtttga tattgacata aaggtcctta taatryggta attcctgatc

18721 agggatctgg aagccatacc tggtrctttm cacaccatac mrtamcatac aaagtgcttt

18781 caagattgca aacttggctt agacctsctt cagtgagctc ctatcctata gtaaaggcag

18841 mtagccaatt attaaaaatg gtcaaggcaa ttgtacttcs aggcagtagt agcaattgct

18901 ctgctacctt gcatcttgaa gtattttcag caasaggatg acctttaggc cacaaattta

18961 gtcagccctt aaggtggmta ttggtttgac ccatattttc atktastcmt ttttctrcac

19021 ttgcctaatc cttctgtgat actgccagtg cttgmcatta saggacctta gggagaccaa

19081 acaggctaga gaggagagac aggagatacc tatatctaat gcttcaggtt aatacttcct

19141 agktttcttt cattgaggat ttctcaacam tttttgtatc ctaccgagag ccttgccctk

19201 tctttcttct caaggacatt ccgagcatgt cagacctgag gactgcaagc agctgtaaca

19261 ggcttcatat tcagcagatc tttccttttg agaatctgga caagctccaa ctaatctmaa

19321 aggatggctt gcaggccacc tggaaaaaaa aggtagtttg aggaactcat trttgtggga

19381 attctgtctt trgctttawr ggcttraact tcctataact ctccttttta aaacagaaca

19441 aaaaacagaa catgagccag ttttcatcaa ttcctatasw ttttttcttt tgcatrtmca

19501 yatayatttt aactttayar atgagttygg cctrtttcat ttatycytca gagctrggct

19561 ccagtgargt ctgtaagggc aagcayactt gatccccaay raagaaygag agatgcaaar

19621 camtawatta yttcytttcw caycwcayag carrawagay tkaayraayy tayrccttyt

19681 gattagyrgs cwttkaaatt attyccactt tyctytggca gaykggtatt aagtkytsar

19741 gatttgttka yrmatarkac yaactymatm trtrttagcy cwgttttggy aggccyaatt

19801 cyattrtcwy tgycatttcy ttstytyaag aaatmaaaak ttyttarytt gaaraacmat

19861 raaattgttr aaaagtagaa taasagagac ccaagggcct gtgtaagcta tttactggat

19921 ccctrgyttt ctgtaymttg tttttccttt tgcatagatt tgcttagctg ttttaytttt

19981 ttwttttyrm agwttcyaaa cygctgsgmt wacrggsstg cmccmcscct gsctagctgt

20041 tgtaataact ggrgttctat atgcctgtaa ccattcttga tttctctgaa tatcctggaa

20101 ctttggtggt accccattta tataagctgt tcaagaaatg taccatgtag attgagtaga

20161 aaacaattct gtttaccttg gcaatattct agcatcgtac tacttaaaat acaaattaaa

20221 agaaggaaat gctacaaaac tagctggaga ggcactttsa ttgaggtgga ttrctcagtt

20281 cttaaaatag trctttataa aagaagcctg aggcaatgtg ragragaatt cttacakaac

20341 tcatagggtc agaccacats ggamcttttc trwgtggctt gatggctctc ttggttgaga

20401 aarytagyty ctccttcyrt tattttcaac cccttgattt cttgamccyc actatatttt

20461 rtgmtragaa cacaagggta ttaamaaccc acmttstcga ggattgctca tcagtagaga

20521 ctggagaata aaacacagta tgggaatttt ggtaattatt cagctctaaa ttgctcttgg

20581 aaatgagggg aagtacacag aatagagctg gaatgaatag aagaatttgc atgtttktyg

20641 ctaagctggt agctagaata taasagctct acacagctct aaatctccac tcctcaatcc

20701 acctgaagaa aagggtaaac atagttsaac tcaaccacta gttactaatt gggacagact

20761 ttcccagtgt actgcatttc aataatttat ttttctttta tctcttttcc agatcttcct

20821 cagaagaata ggcttgttgc tttacagtgt tagtgaccca ttccctttga cgatccctag

20881 gtggagatgg ggcatgagga tcctccaggg gaaaagctca ctaccactgg gcaacaaccc

20941 taggccagga ggttctacca agatactttc ctgggcccag mtaggaagat aaagtctcaa

21001 aaacaaccac cacacatcaa agtgggtaag ctgtccctaa aagcataata attagtcwtt

21061 aattttgatc ttgttttyca gtatacattg cacttagctt tccactgagg ttgtatttat

21121 cattatcawc tatatctgct ttggtaaaaa tagcttccta actaacctgg gaagtaactg

21181 agtgtgaaat gggttaaart gatgatgtra agagagtagc aggctgcatg agttagggtg

21241 tttagmgtgg gactgmagta tgtggtagag acttacagct aaattgtkcw ttcttttagg

21301 agaacatgga crrtaactgc cacatcagtg acactgatcw catgggcaaa tcatcatgct

21361 cgatcctggt ccccaaagtc tcccttgaag ccttatagga gaattttgcc aatcatttac

21421 atacttcaag atgccttggg atacctttgg gataaaacaa raaacaagct gttwgagaag

21481 acaaatgaga ggtttatctt tttatgctat gcttgctgat tgatggaagc ctcataacta

21541 caaatggraa cctgacmaga aatggcacaa agttatctat catcaggcag magctaaaga

21601 accaggacmc tgcatactct aggtcagtga tgagagaggc tgattagggg aatgmaggtt

21661 ggmagataga ggtgaccatt cagtgcagta aatcctgatt gtataggctt tgymcattya

21721 actaaggwat aagrctatga yttrrtggtg tytctagtgr ytcmycaagt rwtattaggc

21781 aagacatttt cctgtttatg ccatagtttc ctattctgtt gaatgaggma rttttmtctc

21841 taargaccta aaagtttcaa ctttatasgt ttctaagttc tgtagagaca ttttctatag

21901 ctcattaatt tgaatcttct cataactcta gcacagtact cagttaatac ttmttcatgg

21961 cacttacatg gtttctttcw trtmtttttt tatagctcct gattgttccc ttywtatcta

22021 ccaaatcatt atccttccca aaagcagtgc agagagctga rycttcagca sgwycaagaa

22081 atgtgaacac accgaaggaa gycagcyttm cmaccwgarg atcaacatgc ctggcatgct

22141 agcattttag aacagcagaa tgaagtaagt tgttggtgtt gcaatcctgt gaggattast

22201 ttagagctaa tagctgtttg ttggaagttg gtgtgggatg aggtataatg atctaatgtg

22261 aagktggggc ccaatatgag atggaaggat gaccagtaac ccatattacc aactgggttc

22321 actgaagcaa ctcaaagata ctatagtctg cttaccagtt gtatcacaga ggaatttagc

22381 ctcaccttgt ctgttctttc tcttcactgc atagatactc tgccattctt agaggcctaa

22441 acacagagaa acctaaaaca aaamcgtttt tgttttttgc tgaaagggtg gccctttctc

22501 atctcctctt gtgagagaat ctgtckkcag ttaagattta mtgttaaagg aaacctagtc

22561 tctgaagcag ccattatact tgtmaaaaat gtgaaaatga agtcaatttt ctaaaggaat

22621 ggggaaaagt gaagatataa aaggagagga ttgactaggt gctmtctaag ggccctttay

22681 kaaaatactc agtcatctgt ggcatcttat ttggctctcc cagaaatcct tgtaaatagt

22741 tgtagcagaa tgttaagagg cwyatacatt gtgtgtttwa aatcmtctgc cactcattag

22801 gtgtatgacc tttgmcaatt tagagtctkt gmttagactt gtctatttgt gaaggttaaa

22861 tgaaatcatg tatgtaaagt gcttagtrca gtgcctggca catgrcaagt aaaaggtaac

22921 ccaagaagtt tcataagttc atttgacaca atacaagtga ccactramac catctagtag

22981 cagrcagagt tggcatgctt tggttctatg taagaaatcc ctaaggtaaa agtttataag

23041 tagaagagca tctgtgttgg tgttgattgt tgtwatratt attattgtta gtaatataaa

23101 taatattggt actaataata gmttattrta attattaatg mcactttttg ttttcttctt

23161 tctgtgatgc ttctcatgcc tcttgtgccc ctcactgtat tttgtctctt ctacttctta

23221 ctttctctga atgtctgcct ttgcttatct cttrcactca aaagtgtgta tttctttgtt

23281 tgtctatttc ttctttgaat ctctttggtc sttcttctat ctaaagtgtg tcttacccat

23341 ttccatgatt ctcttgctag tttcttccmt gtgtaccttt gtctcattta cttttttgtt

23401 cccaagagtg gtctgtgtct tgtcttagat gtatctctct agtttcttca ttttgtttct

23461 aattctcttt gctctcctag atctagctct tctttcactm ttcttcmctt catgtctcty

23521 ttytgmgtca catgctgtgt cccttttgtt catttcttgc tctgcctacc cctctcttct

23581 ctgtctrymt ctmyttyctc yttgtgaact ctgattmttt gtcacccctt ccccttcwyg

23641 ttggtttgac atttcacctt tttctgattc tggccatccc cttctgctgt ttctactctt

23701 tatctcacat ttctcttttc tacatattct ttcctgcctc tcttgggcta ttttctctct

23761 cctcccyyaw kctctgtgtg ccccagtgtc tctttgctct ttgtgacttt ccatttcagc

23821 attcatctct gttctcgtgt ttcttctctg cttcttccct ttctactcac ctttgagtat

23881 ttcagcctct tcatgagtct atgctccccc tctttgattt catgtaattc tctctccttg

23941 catatttctt tgtgtatgta tgtatgtgtg cgcgtgtgtg tgtcatgtgt gacagagggg

24001 cttcctaacc ccttcccaat aggtgcagaa tgtcagctat caaaataagc attgcagagc

24061 tgttccttat gccaggccgc cctgtgagat gatctaagac caaaamaagg tccragrgtc

24121 cagtcagaac tgaagtwgga cagaaagtgg aaggctcata tggatagaag gcccaaagta

24181 taagacagat ggtttgagac ttgtgaccca aagacwarsa tggaaagccc rtgttccaag

24241 atagatacaa gcctcaggcy tgaaaccaac araagsctca agggccaaga wwrmarakgg

24301 tggccykwrt tggaymgaag gcctgagttg gatagaaggc tcaaatcttg agatagaagg

24361 ccaaagacct aggacaggac acctggaagg cctaagaacs gggacctgag acataagaca

24421 aaagacctaa gatgagacca rrryyktrkc tkgaaaacct aaaacccaag gatggaaggc

24481 ccctgrcaca magcctacct agatggacag aaggcccaag agaraaagat atmtcaagac

24541 taamyaggtg ggaarctaga ggcccatgac acagaaccca ggaaggaaag aagccccaag

24601 accaaaggaa atcccaagat gagaacccta aaccccacct cttttctatt sctmtmctcc

24661 ctactcttgg acattttcag ttctcccttt cttcttatat ctccatttaa acctacttct

24721 tttgagatgt actttttgat gttgccattm mccataaaga aacatatctt tagattgaca

24781 atattatgct tgggccagtc ttaagccary tttwatcaca gttttgagcc atttattaag

24841 tttttgattt ttaaacttcc atttctcttc acatcttctc cacttgagag agacamcaaa

24901 atccagtcag tatmtartct grcttttgyt attccctsag garcagayay ycatataggt

24961 gacactgtat ttcagtcctc ccttttcctc agaagcccta ggctgataag amaaggaaag

25021 catcaggctg ttaggggaaa aaaagtgcca ggctatctag agaaaatgtg aagagatgct

25081 ccaggccaat gagaagaatt agacaagaaa tacacagatg tgccagcctg ctgagaagca

25141 ccagccagca acaccttcct tctttgagct taggtgagca ggattcttgg ggtttgggat

25201 tcctagtgat ggttatgaaa rrggwttggg cctgggacaa agtgaggccc caaggagaca

25261 gcmtgaactc cctgctcata gtagtggcct aataatttgg tagactgcac caacactact

25321 cctgggttta atacccacct ctaggcttaa agatgagaga atctgggaat attgagcagg

25381 tttaatcctt tccttgattt ttttctccct myttatatgg gaagataatt taaatraytg

25441 ataatatata tgaaagcact gtaaaacaca gragaaaaac caagacrttc tcagtaatam

25501 tatattggca atcatgcaca gctaacattt gaaagtgcas tgtcagttgt gaagcattat

25561 gtaaatcagg agtcatcaca ccctttctat aaaggatcaa atggtaaata ctttagactt

25621 tgtgggccat atggtctcag tcacatattt ttttttttaa aaaccttttm aaaaggyaag

25681 aatcattctt agyttktgar ccaagtgaat ttggcctgtt ggccatagtt tmataacccc

25741 tgatgtaawt waataccagg gacaatgtgc tgaataatat taatttcctt ctttgtgcct

25801 gtccctatgc taggcactaa ggatgcaatg attattgatg gtcacatcta ggtgacctga

25861 agaaaaatat atgaatgtgc tttgtaaact ataaagcact tgaaagcttm tmagtcagag

25921 ttgatsaatg aatattgatt acatagtarc ystattctac agttctaggc actgtagata

25981 caaagataac agaaggcaga ataaagtgct ttstcaaaag tatataatac tatgcagama

26041 ttaggaattg tttgataaat gaataactca tgtatttgag gccattgtgt ttctgctgct

26101 ctggtaattc tgagtaaaaa tgcagtattc caggtagcag aaaagaaaac acacggaaac

26161 tgacttttaa actataaaat atactacaaa cacaagggat ttaacctatt gtgatcacct

26221 ataatgtgcc agataccatg ctgggcacta cagattatca aagggaaaaa gtattctcat

26281 agaattaaaa atttcagaaa ggtacatatt aaagtgcttt gtaaactaaa gcactataca

26341 aataccaatg ggctacatgt ttatgaatga atgaatrart attaattacc tcttacatac

26401 cagctattgt tttgggtact gtaaaatcca agaktaatgc tcccaagtaa taagaggrat

26461 gtttatgccc tatactattc agatataaag aatgagatat tgcttaattt taatcaatca

26521 agactttact ggtgaggtca agstaaatta ttaccagtac attttccmag gtaacaagaa

26581 aasasgtaat gaatgaratt gtactttcta aagtactaca caagtgtaaa acattagatg

26641 ctttctgtat gaataaaact ttctgtaaga taggcattgt ctctacaaaa ttctcattgt

26701 atgtttgacc cacaatgaga aaggtagtat aaatagttat acaaactgag agtttaaata

26761 cttgttaaat gaatgrgatc aatctcaatt gcctactatg tgaactcact gttataggca

26821 ctaaagagat acacagactt aacatttcat tgtgcctaca gctaaaaata agacaatata

26881 tgttagcatt ttgtgaactc taaggcacca tataagtgta actattgatt ttctcatttg

26941 gtgccgagta ctaggcttrt aaaattgtat cacagttatt atactatgaa aaaagtmgga

27001 aaatttttga amaagaattg ttaactattg aacactcyra atataataca taaaggatta

27061 aatatctgat gaatgcaama ctggataata attatctatt atgtgccaat ctccttgcta

27121 ggcattgggg atgcgaagat aaaccatctt tattgtgtcc tgggtagcag aagaaawtat

27181 atataaaatc aatttataat tcgtaaactg ccatacatat ataagcaata tctgctgaat

27241 gatcattgat tacttacyyt ctcttatccc cagagatagc aactagggat acaaaaattc

27301 attaccatta ttgaacctac aacagagatc tgtgtaaayt ttacaaagcc tacagttcta

27361 tacagatagg aattaactgt tggcttaatg aatgattact gattactttc tawstataar

27421 gctcgaaact gcttatcccc mraaaggata taaaaatgac tttaccatta taaagtgctg

27481 wgtkamasaa ggaaattaag taacataatg tgggatccag agtaaaaatc actaaaccaw

27541 ksctcagctt attcagtgat aaaaacatgg gaaaaatgga attgtacaag gcagtatgct

27601 aaatgctgag ctargtggaa agataaaatc ctgtccagaa ggmtacttgc tccctggcct

27661 gactgaagag atggaaaatt tttgcaaaaa gcaaggtgtt gtmatcctcc atcmgtcttt

27721 taaatgttga atgctgaaga tggaggtgaa ttamatccac cttggcctgg cmtgagraag

27781 taaaggagta aaaataaatg mctcaggttt gcttttagat tcatttgata aacamagcat

27841 cttttatgtg gaatatatca ttctaggtcc tgagsataag agaratgaaa tgggccwagt

27901 ywaragggca ttagatcaaa gattatagct gaagatamaa aaacatcttt gagttrattk

27961 ttaaataaat attttaatgc ctattctctg gaaggtacta tgcttcataa attcgttaaa

28021 taggtctggc ccagaaaacc cactgacttg ccttggagat taaaaaaata gaaaaaaaar

28081 rsacaaggtt ctttcaaaat aaaatgaaga catttctcct agttctagaa agtcccaatg

28141 tcacttcctc attgacctag tttgaagcca gmagactgat aaaaggactc agggtttgtc

28201 ctttaattca ttaactwamy attctgcttt tattacagtt aaatgattta agatgagaat

28261 caagaacaac tagttttaaa satatttgct cattggcctg gcttagagac aggaagammy

28321 wtgagcaata aaaaaatatt ttcctacatt ttccatattt tgtaaaaatt tattaacrst

28381 gaataaagta ctgttcttaa gtgctsarga gaatgaagam atttaaaaga cctaaaccaa

28441 aatgtacttt aatctcatta gctcatggtg gaaacacaag aacaaattta ataagagact

28501 gcagttcttt cattagttga aaaaaaactt yattgagtag ctgtgatatt aagatactaa

28561 gaagaaawgw twaaacaaat cactttctct ttggcatgct ttgtagagta ggaataagag

28621 aggrctttct cttttytcat tcatacaaac attgaagaag atgatactaa atgctaartg

28681 aaatagatct ggtacaaaag gcacttarts tgacttggag atgcagcaaa aamaaaacaa

28741 aamaaaagaa atggagtgaa actcttcatt aaataaaagt gtctattakc tcaaggcatt

28801 ttgttaattc ctagacagaa aacaaaaaag atcatgactg aaagacactt gcttwttwat

28861 tggcttggaa actagaatat argagagagg ttactgttma tttcttcmat ktattcattc

28921 attctacaag tatrtttgag tgcctattmt gtataagctt tttrgctrkr ttcccagttr

28981 gccccagaga catgaaaaaa atgaatgcct tcccagagaa tgcttaawac tttccttttg

29041 gcctgttttc tggttaggga cacggcttag tccctaaata atattgtggg gtttaattcc

29101 tactccatat ctcttmtacc actctggcca ctacaataag caggwagctg ggttttgtag

29161 tgagcttgct ccttaagtta caggaaccct ccttataata gacacttcat ttttmctaac

29221 ccatccctca tgaaaaatga ctgtgwytac cactactggg cagmaggagg gatgakracc

29281 aactaattcc caaaccccag tctcattggt accagccttg agaaaccacc catactcaag

29341 ccacaattgg ttttgaaata catttacmag ttttgtctat tttcagttct ttacmtttta

29401 catgctgaca aatatacact gcctaaatag atctctttca aaaacaatcc tcagatagca

29461 catagcaaaa tggagatgga gacatgattt ctcttttgca acagcttctc aacttamact

29521 ttagaaatct tctcctttty accatcaaaa catgctcaag aagggtttst tatagtagaa

29581 taataccagt ggatgaaaat agcttaacaa ttttaccatg cttaagtttt aagaataata

29641 gaaattgaaa ataattggca aaaattggaa ggaaaaaaaa mcmaaaaatk yctctaaatg

29701 taggcctaac tgggctttga ccttttctat ttttaaatca ctcacagagg gtgggacagg

29761 aggaagagtg aaagaaaagg tcaaacctgt ytctaagggc arcctgcytk tgytytgaat

29821 tggtcttaag agcattacta cctccagatc taactwgggg attgcttttt tcagtttaat

29881 gcaattccaa aaactggtca ttgttgagat gaggacaaag tcctttgtcc ycactaattt

29941 gctatamgtt tttgaaaars atttttsgtc taaatggtta tcaactaaac cttgtgttag

30001 ataagaatgg aatttattar ktsaatcaat gtgacctctc tatcataagg ttgtcttaaa

30061 gctgaagtca maatmtgctr caaaggaaga ggactttatt gttcactgta gttcatacay

30121 actttcaaag catctamact ttagtttaca tagcaagcca atmayrmcat ccrtaaamag

30181 agaaggaaat agaaaaatgr cagaatatrr ttgstggagg gagcmagttt gaagaggatt

30241 tggggttsaa artttctagt ttttrytytg tacattttta kttaaamatc aggtatctga

30301 taatactaat gtttagcttt taatatgtgg tattagctgg actcagtaac acccctttct

30361 tcaggtaggg atggggaatg cattattgga aartggaaag gagaaagtaa ctaaaagcct

30421 tcctttcaca gtttctggca tccyaagact accactactg ataaacaaga ataakagaac

30481 ayyytaccat catctgattt ttrtcacata aatgaagttg tgaacaaatc tgtttttmtk

30541 magmaaaaga aattgcttca tctttaccct tctaccttaa aagraatcta tgccaaaagc

30601 taagataaat ggaagacaya cttggacttg tgaactgatg tgaaatgcag aacatttgag

30661 ccttgggtgt tttgaagatt gaaaaatctt gctcagcatg aatgaccacc aaaaagcaac

30721 ctaaagccat ctasatgtca caactgaaac aaattgggga gtktgttttc taatgtcaaa

30781 taaaatgtac tgttttgaaa rctttgtatt ttgatgtgac aagttctaac tcactgtccc

30841 taaccacact agttacctca rttttttcmt wyctttkrcc tcattttttt mwwwarrcta

30901 ccttaatcac ccagcttggg taaatggttt gaggtctcat ttkcttatag cttcagttgc

30961 tttggtcagg gatactgaga agcaca

//

Figure S1. Nucleotide sequences of eutherian consensus gene *Xist* presented in GENE BANK format.
