# Supplementary material for: A Dual Origin of the Xist Gene from a Protein-Coding Gene and a Set of Transposable Elements
Source: PLoS One. 2008 Jun 25;3(6):e2521. doi: 10.1371/journal.pone.0002521 (PMC2430539; doi:10.1371/journal.pone.0002521)
Supplement: Figure S2 — Alignment of the chicken proto XIC region with the human and mouse XICs. (a) Sequence alignment of part of human Xist intron 3 and a part of Lnx3 exon 3 (ACC XM_420296.1). (b) Alignment of part of human Xist intron 4 and part of Lnx3 exon 5. (c) Alignment of part of mouse Xist exon 5 and chicken Lnx3 exon 5. (d) Alignment of part of the human Xist promoter and Lnx3 exons 1 and 2. (e) Alignment of chicken Rasl11c exon 3 and the 5′-region of Xist. (f) Alignment of chicken Wasf3 exon 1, 2 and the 5′-region of Xist. (g) Alignment of chicken Uspl exon 1, 2 and the 5′-region of Xist, (h) Alignment of chicken Uspl mRNA (ACC NM_001031123.1) and human ENOX EST BC071776. (a–g) computed with SSEARCH34 from FASTA package; (h) computed with WUBLAST 2.0. (0.05 MB DOC) [file pone.0002521.s002.doc]

**A**

G.gallus *Lnx3* exon3 versus H.sapiense *Xist* gene (ACC U80460)intron 3

s-w opt: 142 Z-score: 340.9 bits: 76.8 E(): 3.5e-17

Smith-Waterman score: 142; 59.756% identity (73.171% similar) in 82 nt overlap (615-691:38556-38634)

620 630 640

Lnx3 GGTGTCCTGGTTTCAAGAAATACAAATGTGA

.: :::: ::::.::::::: :::: :

HU-XIST TTTGGTAGGCCTAATTCCATTATCACTGCCATTTCCTTGTTTTAAGAAATCAAAATTT--

38530 38540 38550 38560 38570 38580

650 660 670 680 690

Lnx3 ACTACAACGGAAAAGGAACC----CTGTTTCCAAAGGGAA-GGAAGAGCCCC

:: . . :::::. ::.. .:::: ::. :::: .:.::::::::

HU-XIST –CTTAGCTTGAAAAACAATTGAAATTGTTAAAAAGTGGAATAGGAGAGCCCCGGGGGCCT

38590 38600 38610 38620 38630 38640

**B**

G.gallus *Lnx3* exon5 versus H.sapiense *Xist* gene (ACC U80460)intron 4

s-w opt: 123 Z-score: 280.8 bits: 64.8 E(): 7.8e-14

Smith-Waterman score: 123; 58.678% identity (61.207% ungapped) in 121 nt overlap (40445-40565:1282-1397)

40420 40430 40440 40450 40460 40470

HSXIST GGATGCCATGGTGTATAATACAACAAGTGAGAGGTGTGTCTTTTTATGCTATGGTTGCTG

:::: : ::::::: :: :: : :::

Lnx3 TTGGGAATCAAACTGATCAGGAAGACAGATGAGGCAGGGATTTTTATTCT--GGAT-CTG

1260 1270 1280 1290 1300

40480 40490 40500 40510 40520 40530

HSXIST ATTGATGGAAGCCGCATAAATACAAATGGAAACCTGACTAAAAATGGCACAAAGTTATCT

: :: ::: : : : :::::: :: :::: : :::: :: : : :::

Lnx3 CTAGAGGGAGGTTTGGCAGCCAAAAATGGGAAGCTGAGTCGGAATGACAGAGTCCTGTCT

1310 1320 1330 1340 1350 1360

40540 40550 40560 40570 40580 40590

HSXIST GTCATCAGGCAGGAGCTAAAGAACCAGGACCCTACATTCTCTAGGTCAGTGTTGGGAGAG

: : : : ::::: : : : : :::: :

Lnx3 ATAAACGGCCAGGATTTGAGGCA--AGGAACACCTGAGACCGCTGCACAGATCATCCAGA

1370 1380 1390 1400 1410 1420

**C**

G.gallus *Lnx3* exon5 versus M.musculus *Xist* gene exon 5

s-w opt: 92 Z-score: 182.8 bits: 44.5 E(): 2.2e-08

Smith-Waterman score: 92; 65.672% identity (77.193% ungapped) in 67 nt overlap (35166-35229:1138-1197)

35140 35150 35160 35170 35180 35190

MOXIST ACATCTGGGAAATGTAACTAAACAGTGCAAATGTTTCTTCTAGGAGAAAAGCTGCTGCAA

::: :::: : :: ::::: ::: : :::

Lnx3 CTGCGCCACCCAGGTCCTGTTCTCCACCTCATGGTTCTGCAAG-AGAAAGGCTTTTCCAA

1110 1120 1130 1140 1150 1160

35200 35210 35220 35230 35240 35250

MOXIST TA--GTGGCACTGACCTTCGAGGAAGCCA-TTCTGCTCTATTTGGTTCTCTCTCCAGAAG

:: : :::: : :::: ::: :::::::

Lnx3 TAAGATTGCACAGC------AGGACTCCACTTCTGCTACAAATCAGGAAGTGATCCATGT

1170 1180 1190 1200 1210 1220

35260 35270 35280 35290 35300 35310

MOXIST CTAGGAAAGCTTTGCCAGCTGTTTACATACTTCAAGATGCACTGCTACCCTACTCATGCC

Lnx3 CACCTTGATAAAGAGAGACCGATCAGAGCCCTTGGGAATCAAACTGATCAGGAAGACAGA

1230 1240 1250 1260 1270 1280

**D**

G.gallus *Lnx3* exons 1, 2 versus H.sapiense *Xist* gene (ACC U80460) promoter

s-w opt: 147 Z-score: 356.7 bits: 80.3 E(): 4.6e-18

Smith-Waterman score: 147; 61.111% identity (61.111% similar) in 126 nt overlap (103-218:18280-18389)

110 120 130

Lnx3 GGCTTC-CCTCTGCTCTCCTCTTCCCGCCC

::: : ::::: : : :::: ::::::

HU-XIS TCACAAAGATGTCCGGCTTTCAATCTTCTAGGCCACGCCTCT--TATGCTCTCTCCGCCC

18250 18260 18270 18280 18290 18300

140 150 160 170 180

Lnx3 CC-GTCCATTCCCCTCCAGC----AGAGGAACTCTGTCAGACAGCTGCTACAGGACACAT

: : :: ::::: ::: : :: : ::: :: :::::: ::: :: ::

HU-XIS TCAGCCC---CCCCTTCAGTTCTTAAAG---CGCTG-CAATTCGCTGCTGCAGC-CATAT

18310 18320 18330 18340 18350

190 200 210

Lnx3 TTCCTTCCTGCTGCC----CTGGGAGTGCACTTCCT

::: :: :: :: : :::: :: ::::::

HU-XIS TTC-TTACT-CTCTCGGGGCTGGAAG----CTTCCTGACTGAAGATCTCTCTGCACTTGG

18360 18370 18380 18390 18400 18410

**E**

3>>> - 83 nt versus

vs huxi406500ns.fas library

G.gallus *Rasl11C* ex3 versus H.sapiense Xic locus

rev-comp s-w opt: 309 Z-score: 54.1 bits: 24.3 E(): 0.35

Smith-Waterman score: 309; 63.415% identity (69.512% similar) in 82 nt overlap (2-83:60126-60207)

10 20 30

rasl-- CTCCAGTGAAACAAAGGGAGTATCTTGCACC

: :: : ::: ::: ::::: : ::

huxi40 AAGAGGAGACTGATAGAAACAGCTCTTACATACAAAGCCACAGAGGAGGTATCCTAAACT

466600 466610 466620 466630 466640 466650

40 50 60 70 80

rasl-- TGTAGGGAGATCTGTTCCCCATCTATGGTGAATTTTCTTGAATACAATGCAC

::.:: .::::::: ::: :::: :::: : . ..::::: ::: :::

huxi40 TGGAGATAGATCTGCTCCTCATCCATGGAAAGCCAGAGGGAATATAATACACATGATAGA

466660 466670 466680 466690 466700 466710

**F**

G.gallus *Wasf3* ex1 versus H.sapiense Xic locus

rev-comp initn: 96 init1: 96 opt: 98 Z-score: 71.6 bits: 28.2 E(): 0.24

Smith-Waterman score: 121; 58.333% identity (64.706% ungapped) in 132 nt overlap (131-1:428951-429070)

130 120 110

wasp-- TGAGGCTGCCCAGCTGCTTGATGATGGCAGCC

:: :: : :: : ::::: : :

hu4065 AAGCCCACCAAAGCCACTGGCCACTTACCAAGACTTCACAATGGTTTGATAACTAGGTGC

835420 835430 835440 835450 835460 835470

100 90 80 70 60 50

wasp-- AGCGTGCTG-TTGGTGACACATTCCAGCTCACTCGTCACCCCCTCAGGTAGGGCCCCCCG

:: ::::: :: : : : : : ::: :: :: : ::: : : :: ::: :: :

hu4065 AGGATGCTGATTTGAGCCTC-TCCCAATTCTCTAATAACCTCA-CTGG-AGGTTAACCAG

835480 835490 835500 835510 835520 835530

40 30 20 10

wasp-- GCACAGGTGCCGGGGCTCGATGTTCCTCTTCACCAGCGGCAT

::: :::::: :: : :::::::: ::: ::::

hu4065 GCA---------GGGCTCAATATACCTCTTCAGCAGTAGCATAGTTTCTGATTGAGTGTG

835540 835550 835560 835570 835580

hu4065 GCATCACCTATAGGACAGGGAGAAGAAAAGAAACAAACGTAAGATGGAACAGTCAAGGTT

835590 835600 835610 835620 835630 835640

G.gallus *Wasf3* ex2 versus H.sapiense Xic locus

rev-comp s-w opt: 251 Z-score: 89.8 bits: 31.6 E(): 0.024

Smith-Waterman score: 251; 55.072% identity (58.915% ungapped) in 138 nt overlap (1-135:427610-427741)

10 20

wasp-- CTTCCTCCAC--GGTGGAGTCCAGCTGCGT

:::.:.:.:: . . :.:: .::..::

hu4065 CTGACAAGAATCAGGGCAAAGTAAGCCTCTCTTTCCCTACTTACCTTAATC--ACTATGT

834080 834090 834100 834110 834120 834130

30 40 50 60 70 80

wasp-- CACCTTGATGACC-AGCAGGTCCACTCTCTCCTGCAGCGAGTTCATCCTCATGTAGAAGC

.:::::.:: ::. :: .:..:: :..:::..::: ::. : :::..: :::: : .: :

hu4065 TACCTTAATCACTGAGAGGACCCTCCTTCTTTTGCTGCATGGTCACTCACATGGATGATC

834140 834150 834160 834170 834180 834190

90 100 110 120 130

wasp-- TGTTGGCCTCGTTGAACAGCTCCCCAAAGATGTCCTCAGCGTGCCGGC

:: ::: ::: :.:: .::...:::: :.::::. .:. ::.::

hu4065 TGGTGGACTC--TAAAATACTTTTCAAA--TATCCTTCACAATCCAGCCTAGTGAGAAGT

834200 834210 834220 834230 834240 834250

**G**

G.gallus *Uspl* exon 1 versus H.sapiense Xic locus

s-w opt: 205 Z-score: 65.4 bits: 26.9 E(): 0.53

Smith-Waterman score: 205; 58.462% identity (64.957% ungapped) in 130 nt overlap (6-122:91420-91549)

10 20 30

usp-ex AGTCGGGCGGCTGCGTCCCAACA-TGGCGGCGG--

..:::::::: : :..:: .::: ::::

hu4065 AGGTCAGGAAACTGGGGGAGGGTCCACGGCAACGGCTGCGGCACGGCAACGGCTGCGGCA

497890 497900 497910 497920 497930 497940

40 50 60 70 80

usp-ex CGGC----GCTGGGGC-CGGCGGCGG--GGCGCGGAGCCGCGGCGGGCGGATGGACGTAC

:::: :::::::: ::::..::: ::.::. . . ::::::. : :: : :::

hu4065 CGGCAACGGCTGGGGCACGGCAACGGCTGGTGCATGTTGCCGGCGGAGGCATTTAGGTAG

497950 497960 497970 497980 497990 498000

90 100 110 120

usp-ex TG-ATGACGGTCCGGAGACTCGCCT--CCATCTGTACCATG

:: :.:::::.. :: : : :::: ... ::::. :..

hu4065 TGCACGACGGCTGGGCGCCGGGCCTGTTTGGCTGTGACGCCCACCCCCGGCTTTCATCCG

498010 498020 498030 498040 498050 498060

hu4065 CCTATGCCCTAGGGCTAGTGGAAGACTTAAGATGGCGGCGTTTGCACGGAGTGCAATCAC

498070 498080 498090 498100 498110 498120

G.gallus *Uspl* exon 2 versus H.sapiense Xic locus

s-w opt: 145 Z-score: 350.4 bits: 78.1 E(): 1e-17

Smith-Waterman score: 145; 62.121% identity (77.273% similar) in 66 nt overlap (12-77:18578-18642)

10 20 30 40

usp-ex GGCGCCAATGCCTCAGCTTTGGAGAAAGAGATTGGTCCTGA

::: ::.:.:::::.: ::::..: :

hu497k CCTTGTTTGGCGTGGGTTTTCACCTTTTCACTCTTCTCTAGAGAAGGCCTGTGGTTTTCA

515550 515560 515570 515580 515590 515600

50 60 70 80

usp-ex ACAGTTCCCTGTGAATGAGCATTACTTTGGCTTGGTCAAT

.: :.::::::: :: : : ::: ::: ..:::.:

hu497k GCTTTCCCCTGTGCATTA-CTTTAATTTTATTTGATGTTTAGTCTGTGGTTGGGGCGAAA

515610 515620 515630 515640 515650 515660

**H**

G.gallus *Uspl* mRNA (ACC NM_001031123.1) versus human ENOX EST BC071776

Query= uspl RCJMB04_7a13 gi|71895568|ref|NM_001031123.1| Gallus gallus

similar to ubiquitin specific protease 12; ubiquitin hydrolyzing enzyme 1

(LOC422322), mRNA (5355 letters)

>BC071776 .1| Homo sapiens hypothetical LOC554203, mRNA (cDNA clone

IMAGE:4604595) Length = 1692

Plus Strand HSPs:

Score = 160 (46.7 bits), Expect = 1.2e-06, P = 1.2e-06

Identities = 138/238 (57%), Positives = 138/238 (57%), Strand = Plus / Plus

Query: 361 GAAGAAGGTGGGAGTC--GTCCCACCCA-A-GAAATTTATTTCCCGATTGAGGAAAGAAA 416

| || ||||| ||| | | || ||| | || | | || |||| | | | |

Sbjct: 1042 GGAGCAGGTGAGAGGCTGGGGCCTGCCAGATGATCATGCTGGGCCCATTG-GAATTGTTA 1100

Query: 417 --ATGAATTGT-TTGATAATTACATG--CAGCAGGATGCACATGAATTCCTAAACTACCT 471

|| || || ||||| ||||| | | |||| | ||||| | || || | |

Sbjct: 1101 CCATCAAGGGTGTTGATTCCAACATGTGCTGTGGGATCCCCATGAG---C-AATCTCCGT 1156

Query: 472 -ACTTAACACT-ATTGCTGACTTGCTACA-AGAAGAGAAAAAGCAGGAGAAGCAGAATGG 528

|||| || | ||| || | | || ||||||| ||||| | | || || | ||

Sbjct: 1157 GACTTTCCAGTCATTAA-GATTAGGCACCGAGAAGAGGAAAAGGAATAAAATCAAACTG- 1214

Query: 529 CAAACTCCAGAATGGCAGCATTGAAAGTGAA--GAAGGA-GACAAGAC-TGAT-CTGA 581

| | | | | ||| | | || |||| | | || || ||| | ||| ||||

Sbjct: 1215 -ATATTTCTGGCTGGGAACCA-GATGCTGAAATGGATGAAGAGAAGTCATGAAACTGA 1270
