# Supplementary material for: A Dual Origin of the Xist Gene from a Protein-Coding Gene and a Set of Transposable Elements
Source: PLoS One. 2008 Jun 25;3(6):e2521. doi: 10.1371/journal.pone.0002521 (PMC2430539; doi:10.1371/journal.pone.0002521)
Supplement: Figure S3 — Homology of the mammalian consensus Xist gene exons 2, 4, 9, 10 and various transposable elements. (0.03 MB DOC) [file pone.0002521.s003.doc]

**Consensus *Xist* exon 2 (m2-ph2) versus L3 CR1**

>>L3 CR1 (4489 nt)

s-w opt: 198 Z-score: 89.5 bits: 26.8 E(): 2.8

Smith-Waterman score: 198; 57.576% identity (75.758% similar) in 99 nt overlap (13512-13604:3431-3526)

13520 13530 13540

conxis GGATGAATTCGGAGCTGGTTCTGTACCCCC

.:.::.::::. :::::::: ..: :. .

L3 GNGAAATATGGGCTGGATGNTAGNNCAATTAGGTGGATTCATAGCTGGTTGAACAACTAT

3410 3420 3430 3440 3450 3460

13550 13560 13570 13580 13590

conxis ACTCAAGAGG-----AAGGATGGATCAATTTTAGG-TGGAGTGAAGCCTGCACTAGACAG

::.::::::: : .:::::::.:: :.:. ::::: ::...:: .: :.:: :

L3 ACCCAAGAGGTGTTGATTAATGGATCGATGTCAACCTGGAGGGAGATCTCTAGTGGAGTG

3470 3480 3490 3500 3510 3520

13600

conxis CATCCAAAG

::: ::

L3 ---CCACAGGGCTCTGTCCTTGGCCCTGTCCTGTTCAACATTTTTATCAATGACTTGGAT

3530 3540 3550 3560 3570

**Human *Xist* exon 2 (h2) versus L1MC3 LINE**

>>L1MC3 L1 Eutheria (2487 nt)

rev-comp initn: 206 init1: 146 opt: 194 Z-score: 211.0 bits: 47.9 E(): 2.7e-07

Smith-Waterman score: 194; 79.688% identity (87.931% ungapped) in 64 nt overlap (64-1:2209-2266)

60 50 40

hsex2- CAAGAGGAGCCTAAGGAGACATGACTACTA

::::::::::::::::::::::::: ::::

L1MC3 AACAAGGAAAGTCTGAGAAACTGTCACAGCCAAGAGGAGCCTAAGGAGACATGACAACTA

2180 2190 2200 2210 2220 2230

30 20 10

hsex2- AGGACACATGCAGCGTGGTATCTTCAATGGGATC

: ::: :..::::::::.: .::::::::

L1MC3 A------ATGTAATGTGGTATCCTGGATGGGATCCTGGAACAGAAAAAGGACATTAGGTA

2240 2250 2260 2270 2280 2290

**Consensus *Xist* exon 4 (m3-h3) versus L1 LINE**

Query= consensus Xist ex4 (138 letters)

>L1 L1 Length = 5403 Plus Strand HSPs:

Score = 121 (24.2 bits), Expect = 0.70, P = 0.50

Identities = 85/143 (59%), Positives = 85/143 (59%), Strand = Plus / Plus

Query: 2 ACATTCCGAGCAT-GTCAGACCTGAGGACTGCAAGCAGCTGTAACAGGCTTCA-TATTCA 59

|| ||||| | | | || ||||||| | |||||| | | ||| ||| |||| |

Sbjct: 4847 ACTATCCGATCTTTGACAAACCTGAGAAAAACAAGCAATGGGGAAAGGATTCCCTATTTA 4906

Query: 60 GCAGATCTTTCCTTTTGAGAATCTGGACAAGCTCCAACTA-AT--CTMAAA--GGATGGC 114

| || | | | || || |||| | ||| | || | || ||| |||| |

Sbjct: 4907 ATAAATGGTGC--TGGGAAAA-CTGG-CTAGCCATATGTAGAAAGCTGAAACTGGATCCC 4962

Query: 115 TTGCAGGCCACCTGGAAAAAAAA 137

|| | | |||| | |||||

Sbjct: 4963 TTCCTTAC-ACCTTATACAAAAA 4984

**Consensus *Xist* exon 9 (pm7-h7) versus ERV2**

Query= con50ex7 (151 letters)

>MERVK1_I ERV2

Length = 2555 Plus Strand HSPs:

Score = 107 (22.1 bits), Expect = 0.10, Sum P(2) = 0.096

Identities = 43/67 (64%), Positives = 43/67 (64%), Strand = Plus / Plus

Query: 82 CTCAT-TGGTACCAGCCTTGAGAAACCACCCATACTCAAGCCACAATTGGTTTTGAAATA 140

||||| || || ||||| || |||| || ||| |||||| || | | | | |

Sbjct: 2463 CTCATATGCTAAAGGCCTTCCTAATCCACTCAGCCTCCAGCCACTGTTTGCTCT-AGAGC 2521

Query: 141 CATTTAC 147

||| | |

Sbjct: 2522 CATGTGC 2528

Score = 85 (18.8 bits), Expect = 0.10, Sum P(2) = 0.096

Identities = 53/94 (56%), Positives = 57/94 (60%), Strand = Plus / Plus

Query: 1 CCATCCCTCATGAAAAATGACTGTGWYTACCACTACTGGGCAGMAGGAGGGATGAKRACC 60

|| | ||| |||||| | | ++||| |||| ||| | ||||| || |++|

Sbjct: 1414 CCCTGACTCCAGAAAAACTCCAGGCACTACAACTATTGGTTA--AGGAGCAATTAGAAA- 1470

Query: 61 AACTAATTCCCAAACC--C-CAGTCTCATTGGTA 91

|| || | | || | ||||| | ||| |

Sbjct: 1471 AAGGAAATATAGAGCCATCACAGTCACCCTGGAA 1504

**Consensus *Xist* exon 10 (m8-h8) versus DNA transposons**

>hAT-10_XT hAT

Length = 11,144 Minus Strand HSPs:

Score = 160 (30.1 bits), Expect = 1.2, P = 0.69

Identities = 143/239 (59%), Positives = 146/239 (61%), Strand = Minus / Plus

Query: 229 TCAAATGTTC-TGCATTTCACATCAGTTCACAAGTCCAAGTRTGTCTTCCATTTATCTTA 171

| |||| ||| || ||||| ||| |||| | | || | | | ||| ||||| || |

Sbjct: 9129 TAAAATATTCATGGTTTTCA-ATC--TTCAACATTTGAATTTTAT-TTC-ATTTAACT-A 9182

Query: 170 GCTTTTGGCATAGATTYCTTTTAAGGTA-G-AAGGGTA-A-AG-ATGA-AGCAATTTCTT 117

|| | || ||| ||| ||| | | || || | || || || | ||| |

Sbjct: 9183 -CTGATCAGAT-GATA-CTTG-AAGTTGTGTAACATTACATAGTATTTTAGTAGATTCAT 9238

Query: 116 TTKCTKMAKAAAAACAGATTTGTTCACAACTTCAT-TTA-TGTG--AYAAAAATCAGAT- 62

| |+ | | ||| | || | | | ||||| ||| |||| |+| ||| ||

Sbjct: 9239 TACATTTACACAAAAATATACAGT-ATA--TTCATGTTAATGTGGGATACAAAGCACCCA 9295

Query: 61 GATGGTARRRTGTTCTMTTATTCTTGTTTATCAGTAGTGGTAGTCTTRGGATGCCAGAA 3

||||||| ||||| +| ||| | || || ||| |||| || || | | || || |

Sbjct: 9296 GATGGTATC-TGTTC-AT-ATT--TATT-ATTAGTTGTGGCTGTGTTTGTAAGCTAGTA 9348

>ZOMBI DNA transposon

Length = 2806 Plus Strand HSPs:

Score = 144 (27.7 bits), Expect = 7.5, P = 0.9995

Identities = 75/116 (64%), Positives = 76/116 (65%), Strand = Plus / Plus

Query: 178 AATGGAAGACAYACTTGGACTTGTGAACT-GATGTGAAATGCAGAACATTTGAG-CCTTG 235

|||| | | +|| | ||| |||| | ||||| | ||| || || | | |||||

Sbjct: 1085 AATGCAGCAGGCACGCATAAGTGTAAACTTGCTGTGATAGGCAAAAGCTT-GCGTCCTTG 1143

Query: 236 GGTGTTTTGAAG-ATTGAAAAATCTTGCTCAG-C-ATGA--ATGACCACCAAAAAG 286

|||||| ||| | |||| |||| | ||| | || | ||| | | |||||||

Sbjct: 1144 C-TGTTTTCAAGGAGTGAATT-TCTTAC-CAGTCCATTATTATG-CTAACAAAAAG 1195
