# Supplementary material for: A Dual Origin of the Xist Gene from a Protein-Coding Gene and a Set of Transposable Elements
Source: PLoS One. 2008 Jun 25;3(6):e2521. doi: 10.1371/journal.pone.0002521 (PMC2430539; doi:10.1371/journal.pone.0002521)
Supplement: Figure S4 — Homology of consensus monomer sequences of the main blocks of tandem repeats in Xist gene and various transposable elements (0.03 MB DOC) [file pone.0002521.s004.doc]

**Monomer of consensus tandem repeat A versus Endogenous Retrovirus**

>>ERVB5_3-I_RN Endogenous Retrovirus Rattus norvegicus (8551 nt)

s-w opt: 98 Z-score: 97.9 bits: 27.6 E(): 0.67

Smith-Waterman score: 98; 74.074% identity (74.074% ungapped) in 27 nt overlap (1-27:5998-6024)

10 20

repA GCCCATCGGGGCCKTGGATACCTGCTTT

:::: :.::::::.::.:.:.: ::::

ERVB5_ GACCTCTGTAAATTAGCCCTCGGGGCCAGGGCCCCTTGGGGCCTTGAACATCAGCTTGAG

5970 5980 5990 6000 6010 6020

ERVB5_ TTACAAGTAGCCCCAGAGGATAACATGCCCCACACTCAGAGCCAACAAACCCACAATTTG

6030 6040 6050 6060 6070 6080

**Monomer of consensus tandem repeat B (CCCCAG)n versus Endogenous Retrovirus**

>>ERV18_MD_I ERV1 Monodelphis domestica (6250 nt)

s-w opt: 162 Z-score: 91.2 bits: 26.6 E(): 2.2

Smith-Waterman score: 162; 70.175% identity (83.333% ungapped) in 57 nt overlap (1-48:2354-2410)

10 20

repB CCCCAGCCCCAGCCCCAGCCCCA-------

::::: :::::::::: :::::

ERV18_ TCTAGACCTCACCCATCCTACTCCTCTCCACCCCACCCCCAGCCCCTCCCCCATTCCTCT

2330 2340 2350 2360 2370 2380

30 40

repB --GCCCCAGCCCCAGCCCCAGCCCCAG

.::::: ::::::::::: ::::..

ERV18_ CCACCCCACCCCCAGCCCCACCCCCGATCCTTTCACTCTGCCTCCCCACCTGCTGACCAG

2390 2400 2410 2420 2430 2440

**3-mer of consensus tandem repeat F versus DNA transposones**

>>HAT2_MD hAT Monodelphis domestica (3298 nt)

rev-comp s-w opt: 159 Z-score: 80.0 bits: 24.3 E(): 17

Smith-Waterman score: 159; 64.000% identity (65.753% ungapped) in 75 nt overlap (1-75:1057-1129)

10 20 30

repF TCTGCCATGATGTCCACGTGGCAAATCTGC

::..::: ::: :::: :::..:. :::

HAT2_M ACCAGTGTGAGCCTGAGGTAAAAGTGGAGTTCCACCAATATGGCCAC-TGGTGAGGCTGA

1030 1040 1050 1060 1070 1080

40 50 60 70

repF CATGATGTCCACGTGGCAAATCTGCCATGATGTCCACGTGGCAAA

::::::: :: ::: ::. :::: ::::: :::. . ::.:

HAT2_M AATGATGTGGAC-TGGGAAGGCTGCATTGATGGACACAGATCAGACTGCATTGATGGGCA

1090 1100 1110 1120 1130 1140

**Monomer of consensus tandem repeat F versus DNA transposones**

>>Birddawg_I Gypsy Gallus gallus (5334 nt)

rev-comp s-w opt: 81 Z-score: 75.7 bits: 22.6 E(): 19

Smith-Waterman score: 81; 75.000% identity (75.000% ungapped) in 24 nt overlap (2-25:4773-4796)

10 20

repF3 TCTGCCATGATGTCCACGTGGCAAA

:. :::: :::::: .::::::.:

Birdda TCAACACTCCGGTCTTCATTGCTAAAGGGACCCCCATCATGTCCCTGTGGCAGATCAGGA

4750 4760 4770 4780 4790 4800

Birdda CACCCCCTTTGGTGCCTGATATAGTTATGCAGCCGCAGACATCTGGCCAGAAGGTATGGT

4810 4820 4830 4840 4850 4860

**Monomermer of consensus tandem repeat H versus L1 LINE**

>>L1-7_MD L1 Monodelphis domestica (3400 nt)

s-w opt: 108 Z-score: 83.2 bits: 23.7 E(): 11

Smith-Waterman score: 108; 72.727% identity (77.419% ungapped) in 33 nt overlap (1-31:3323-3355)

10 20

repH AGTTTTCATG--TTTGCTTCCTTAAAATTC

:.::::.::: ::::.:: .::::::::.

L1-7_M AAACAATGTGGAGTTTGTGGTTTTGCACATAATTTTTATGCATTTGTTTATTTAAAATTT

3300 3310 3320 3330 3340 3350

30

repH CTTAA

.::

L1-7_M TTTTTGTTGGTGGTGGTGGTTACTAAGTATATAATAAAAAAATGGAAA

3360 3370 3380 3390 3400

**Monomer of consensus tandem repeat C (109 bp) versus Endogenous Retrovirus**

>>ERVB4_3-I_MM Endogenous Retrovirus Mus musculus (8574 nt)

s-w opt: 220 Z-score: 76.0 bits: 25.5 E(): 11

Smith-Waterman score: 220; 58.824% identity (68.627% ungapped) in 119 nt overlap (1-109:3748-3859)

10 20

repC TGCTAAAAATAAGTTGTCC--ATTGCTCAT

:::.::::::: :: :.:: ::...: ::

ERVB4_ CCACAAGGAATGGCTAATAGCCCCACCTTGTGCCAAAAATATGTGGCCCAGATCATTGAT

3720 3730 3740 3750 3760 3770

30 40 50 60 70 80

repC CCTATAAGACTGAGATACC---CTGTCTACCTCTAGCATTGC-----TGATCTTCAGTAC

:: :::::: : : : .: :::.:::. :. :::::.: ::::.: : ::

ERVB4_ CCAATAAGAGGGTGCTTTCCCACTGCCTATATTGTGCATTACATGGATGATTTAC--TAA

3780 3790 3800 3810 3820 3830

90 100

repC TGACTACCTAAGTCACCATTTTCAGTTAA

:..::::: ::: :::: ::.. ::

ERVB4_ TAGCTACCAAAG-----ATTTACAACAAACCCATGAGATTGCCCAAATAGTAGTTGCTGC

3840 3850 3860 3870 3880 3890
