# Supplementary material for: A Dual Origin of the Xist Gene from a Protein-Coding Gene and a Set of Transposable Elements
Source: PLoS One. 2008 Jun 25;3(6):e2521. doi: 10.1371/journal.pone.0002521 (PMC2430539; doi:10.1371/journal.pone.0002521)
Supplement: Figure S5 — Nucleotide sequences alignment of cDNA of chicken and opossum Lnx3 genes (Mlnx3 and Clnx3) and human and bovine Xist genes. (0.04 MB DOC) [file pone.0002521.s005.doc]

Md_lnx3 GGCTTGAGGAAATACCAAGCCAAAATGGTGGAGGAAAAATGTCAACAGGACCAAAAGAAA

Gg_lnx3 ------------------------------------------------------------

Hs_xist ------------------------------------------------------------

Bt_xist ------------------------------------------------------------

Md_lnx3 GAAGAAGCGCCCCTGGAGCCAGAAGCCAGCAGTCCTTGTGGGGAGACCTTCTTTGATATT

Gg_lnx3 ------------------------------------------------------------

Hs_xist ------------------------------------------------------------

Bt_xist ------------------------------------------------------------

Md_lnx3 GATGCCATTTTATCGATGGACTCGGAGTATTCCTCTGTCACCCCAATGGTGCCCTGGACT

Gg_lnx3 ------------------------------------------------------------

Hs_xist ------------------------------------------------------------

Bt_xist ------------------------------------------------------------

Md_lnx3 AGCCCAGAGCCTGGCTTGGTGAACCCAGCCTTTGAGGAGACAGATGAAGATTGCATCCTG

Gg_lnx3 ------------------------------------------------------------

Hs_xist ------------------------------------------------------------

Bt_xist ------------------------------------------------------------

Md_lnx3 AATCTTTCCACATCTGACCTGACTCGTGGTAACAATCTTGTGGCTGAAACTGTCATTGTG

Gg_lnx3 -------------------.TC...AGA.AGCT.GC.C............GAG..CAA.A

Hs_xist ----------------.T..TC...AGAAG..T.GG.....T.T.T.C.G...T.G..CA

Bt_xist ----------------.T..AC...AAAAG..T.GG.C...T.T.T.C.G...T.G..-A

Md_lnx3 GAAATTTCCCGGGATAACCCTGACAAAGAGCTGGGGATGAGGATCGTGGGGGGCAAGGAC

Gg_lnx3 ......CA...A..AG.T..A..GG.......T........A..A..T.....T......

Hs_xist TCC...C..TTT..CG.T..CT.GGTG...A..............C.A....AA...CT.

Bt_xist CCC...C..TTT..CG.TT.CT.GGTG...A..............C.A....AA.GATT.

Md_lnx3 ACCCCACTGGGTAACATTGTTGTACAAGAGGTCCACCAGGACACCCCCCTGGGCATGGAT

Gg_lnx3 ..G.....A..A...........T..G..A...TTG.G...TT.TGT.A.T.CTGCA...

Hs_xist ..T........C....ACCC.AGG..G.....T.GT..A..T..TTT.....T.CCA...

Bt_xist ..T......A.C....ACTC.AGG..G.....TATA..A..TT.TTT.......CCA.T.

Md_lnx3 GGAAAAGTGGCCCCTGGAGACCACGTATTAGAGGTCAACGGAGTCAACATCAGCAATGTC

Gg_lnx3 ....G.A.A..A.....G.....TA.TC.T...---------------------------

Hs_xist A.G..GA.AAAGT..CA.A.A..ACC.CC.------------------------------

Bt_xist AAG..G...AAG...CA...-..ACC.CC.------------------------------

Md_lnx3 ACCCACTCTCAGGCGATCTCCCTGCTCCGCCATGCAGGGCCTGTGCTACAGCTGGTGATC

Gg_lnx3 ------------------------------------------------------------

Hs_xist ------------------------------------------------------------

Bt_xist ------------------------------------------------------------

Md_lnx3 TTGCAAGAGAAGGGATTCCTGAACAAGCCACCCCAAGACGACAGCCCAATTCCCAGCAGG

Gg_lnx3 ------------------------------------------------------------

Hs_xist ------------------------------------------------------------

Bt_xist ------------------------------------------------------------

Md_lnx3 GCTGGGGAGATTGTGCATGTCACCCTGATGAAGAAGGACCGGCTGCAGCCCCTGGGTATC

Gg_lnx3 -----------------------------------------------....T....A...

Hs_xist -----------------------------------------------.G.TTG..A.GC.

Bt_xist -----------------------------------------------CAAAACAAAA.A.

Md_lnx3 AAACTGATCCGTAAGGCTGGCAAGGCTGGCATCTTCATTCTTGACCTGCTGGATGGTGGC

Gg_lnx3 .........A.G...A.A.ATG....A..G..T..T.....G..T.....A..G..A..T

Hs_xist .TGG..TATAA..CAA.AA.TG...TGT.TC.T..T..G..ATGGT..........AA..

Bt_xist ..G...T.TGAG...ATAAAAG..AT.TATC.T..T..G..CTG......AAT...AA..

Md_lnx3 CTGGCAGCCAAGAACGGAAAGCTGAGCCAAAATGACAAAGTACTCTCTATCAATGGTCAG

Gg_lnx3 T..........A..T..G........T.GG.......G...C..G.....A..C..C...

Hs_xist .GCAT.AAT.CA..T.....C....CTA......G..C.AAGT.A...G...TCA.G...

Bt_xist T.CA..A.T.T...T..G.--------------.G..C.AAGT.G.......TCA.A.T.

Md_lnx3 GACCTGAGACAGGGGACACCAGAGACAGCAGCCCAGATCATTCAGACCAGTGAGAGCCGA

Gg_lnx3 .GGACACC.G..ACAG..G.CC...TC---------------------------------

Hs_xist ..G..A.AGA.CA....C.T.C.TT.T---------------------------------

Bt_xist C.T..A.AGA.CA.C....TGTGT..T---------------------------------

Md_lnx3 GTTCACTTTGTGGTCCTGAGGACTCGAGGCAGCCCAACCATGGAGATGGAGACAGACTGC

Gg_lnx3 ------------------------------------------------------------

Hs_xist ------------------------------------------------------------

Bt_xist ------------------------------------------------------------

Md_lnx3 AGCAGCTCAAGGCCCAGCAGAAGCAGTGGCAACAATGGCACCAGCAACCACATCAGCCTC

Gg_lnx3 ------------------------------------------------------------

Hs_xist ------------------------------------------------------------

Bt_xist ------------------------------------------------------------

Md_lnx3 AGCCATGGGAACAGCCCTGCCCCAATTTCAGTTCCTCAATGGAAGCCAGAGCTGGGCTAC

Gg_lnx3 ------------------------------------------------------------

Hs_xist ------------------------------------------------------------

Bt_xist ------------------------------------------------------------

Md_lnx3 TACCCAAGGCCTCTGGCTTTCTACAAGGATCCCCCTGCCGGCTTCCTGAGCCAGGAACGG

Gg_lnx3 -----------------------------ATTA...C.A..A.ATG.A..T..T...AA.

Hs_xist -----------------------------..T..A..TT..TC----AG..TG.TCT.AA

Bt_xist -----------------------------..TTGGG.AT..TC.TG-ATC..T.TCT.CT

Md_lnx3 ACAATCACCATAAGGAAAGATGTGAAAGAATCCCTGGGCATCACGATTGGGGGTGGCCGG

Gg_lnx3 ...G.TG.AG...A...G..ACCA..G........A..A..A..A.....A.....A..A

Hs_xist ..TCCTGA.C.C...TG.TCC.CCC..ACC....AAA.TGCTGG....AT...C.--T.A

Bt_xist G-T.CAGTGTC.T.A.TCTCCA.CC.TAGT..ATCA....CTCTTTC.ATCA.AACTA.T

Md_lnx3 GAAGGCAAACACAGCGTACCCATCTATGTGACCAGTGTGCAGCCCGTGGGCTGTCTTTGC

Gg_lnx3 ..TAA....A...AGC.C..T..A.....A..A..C..A......A.T..G..C..C.T.

Hs_xist .CCAC.GCG.C.G..AGTT.TGGTCT.-.A..T.AG..AT.AGG.TAT.A...GTAG..G

Bt_xist CCCTTA..T.TATTTACTT...CTGTATAAT.T.AG.GATTTGATT.A..TCA.ACC..A

Md_lnx3 CGGGACAGCCGCATTCAGAGAGGTATCATCCTCCTAAGTATCAACGGCATCGACTTGACA

Gg_lnx3 A....TG..A.A..CA..C....AGATG.A..TT.G.....A..T..G..T..T.....T

Hs_xist T.TCT.TAGT.-.C...TCA..-.GAT..TTGG.A.GAC..TTT.C-...TT.TGCC.GT

Bt_xist AT..T.TAGT.GT...CCCA..-...T..TAGG.A.GAC..TTT.C-TG.TT.TGCC.TG

Md_lnx3 TCCCTCTCCTACCAAGAGGCAGTGACAGTTCTAAAGTCACAGGCAGCCTCGACCATCATT

Gg_lnx3 CAT..GAA....T.T..A..T..CT...CG..G..A..TA.T.....T..CCA.TCAG.C

Hs_xist .T...A.T..GTTG.....A.A.TTTCTC.......A.CT.AA-..TT.T...T-.T..A

Bt_xist .TT.CTATTCTGTTGAGA.GCAGTTT.TC.....G.A.CT.AA-..TG..A..T-.T..A

Md_lnx3 GTCCTGAAAGCCTTGGACATCCTGGTGCCAGCTATGAGCTTGGAGCCTTGGGCAGGTGTT

Gg_lnx3 A.A.....G........A.....TTCAA...ACCCC.---------------...A.CCC

Hs_xist .GTT.C....TTC..A....TT.C-.ATTGCT...T.AT...A.-T...AT.T.AC.C.A

Bt_xist C.TT.CT...TTC......GTT.C-.ATAGCTC..T.AT....GT.T.CCCAT.AC.C.A

Md_lnx3 CGAGAGTCGCCCATGGAGTACGGTCAGAGCTGGTCTCCCCTCTGGCTGGCCTGGCTGGGG

Gg_lnx3 TCTCT.GACAT..A....CGA..ATTC..................A.CA.G.....T..A

Hs_xist GC.C...-A.T..ATATT..T..CTTT.CA....T.AT.TCA..-----TT.TTT.ATA.

Bt_xist GC.C...AAGTT.ATACT..T..ATTT.CA....T..TTTCT..TT---TTCTTT..TA.

Md_lnx3 CTCCCCAGCGCCCTCCATTTATGCCAAGATGTTGTGCTGCATAAGGACACAGAGGAGAGC

Gg_lnx3 T.G..T...TA...T..C.GC..T......A....C..TAGC..A.G..ACC....A...

Hs_xist ...TT..TT.TT.C---.A.C......ATCA..A.A..T.C..CAAG..GT.CA......

Bt_xist ....TGGTT.TT.C....A.T......ATCA..A.CT.T.CCTGAAGT.GT.CAA.....

Md_lnx3 TGGGGCTTCAGCATTGTGGGGGGCTTCGAGGCCTCCAAAGGCAACCAGCCCTTCTTTATC

Gg_lnx3 .................T..A.....T....AGAG......A..............C...

Hs_xist ..A.T........GGTCCAA.AAA..T..ACA.A.TG....A.GT.....T.C.CACC.G

Bt_xist ------------------AA.AAA.GT..ACA.A..-....-------------------

Md_lnx3 AAGACCATTGTGCCTGGCACCCCAGCCTTCCGGGATCGAAGACTGAAATGTGGTGACGAG

Gg_lnx3 ..A.....C........G..G..T....G...CAGCGC...G.....-------------

Hs_xist ....T..ACA..........T.T...AC.TGA....A.CT..A....-------------

Bt_xist ....T..ACA......CA.TG.T...A..TTA.A..A.C...A....-------------

Md_lnx3 ATCGTGGCCGTCAATGGGACTCCTGCCACTGGCATGAGCAATG

Gg_lnx3 -------------------------------------------

Hs_xist -------------------------------------------

Bt_xist -------------------------------------------
