# Supplementary material for: A Dual Origin of the Xist Gene from a Protein-Coding Gene and a Set of Transposable Elements
Source: PLoS One. 2008 Jun 25;3(6):e2521. doi: 10.1371/journal.pone.0002521 (PMC2430539; doi:10.1371/journal.pone.0002521)
Supplement: Figure S6 — Minimum evolution trees constructed using (a) synonymous and (b) nonsynonymous substitutions (modified Nei-Gojobori method, p-distance) [18]. The numbers for the interior branches refer to the bootstrap values with 1,000 pseudoreplicates. (0.03 MB DOC) [file pone.0002521.s006.doc]

(a)

(b)

Figure S6. Minimum evolution trees constructed using (a) synonymous and (b) nonsynonymous substitutions (modified Nei-Gojobori method, p-distance) [25]. The numbers for the interior branches refer to the bootstrap values with 1,000 pseudoreplicates.
