# Supplementary material for: A Dual Origin of the Xist Gene from a Protein-Coding Gene and a Set of Transposable Elements
Source: PLoS One. 2008 Jun 25;3(6):e2521. doi: 10.1371/journal.pone.0002521 (PMC2430539; doi:10.1371/journal.pone.0002521)
Supplement: Table S1 — (0.04 MB DOC) [file pone.0002521.s007.doc]

**Table S1. Tandem Repeat (TR) composition of the *Xist* gene in *Canis familiaris* (C.f.), *Bos taurus* (B.t.), *Homo sapiens* (H.s.), *Pan troglodytes* (P.t.), *Rattus norvegicus* (R.n.), *Mus musculus* (M.m.), *Microtus rossiaemeridionalis* (M.r.), and eutherian consensus (con.) *Xist* gene.**

| **Species** | **Gene**  **length**  **in bp** | **RNA**  **length**  **in bp** | **Exon 1**  **size**  **in bp** | **TR**  **length**  **of exon 1**  **in bp** | **TR of**  **exon 1**  **in %** | **Total length of**  **TR**  **in bp** | **TR**  **in RNA**  **in %** | **TR**  **in gene**  **in %** |
| --- | --- | --- | --- | --- | --- | --- | --- | --- |
| **C.f.** | **37592** | **21404** | **15480** | **5490** | **35,47** | **6191** | **28,92** | **16,47** |
| **B.t.** | **34934** | **24477** | **18693** | **14637** | **78,3** | **15309** | **62,54** | **43,82** |
| **H.s.** | **32063** | **17225** | **11333** | **7138** | **62,98** | **7832** | **45,47** | **24,43** |
| **P.t.** | **32050** | **17189** | **11316** | **3017** | **26,66** | **3742** | **21,77** | **11,68** |
| **R.n.** | **22898** | **15301** | **9430** | **5350** | **56,73** | **6749** | **44,11** | **29,47** |
| **M.m.** | **22786** | **15221** | **9483** | **5397** | **56,91** | **6748** | **44,33** | **29,61** |
| **M.r.** | **21161** | **13490** | **7939** | **4689** | **59,06** | **5703** | **42,28** | **26,95** |
| **con.** | **30297** | **17561** | **11506** | **3419** | **29,71** | **4161** | **23,69** | **13,73** |
