# Supplementary material for: A Dual Origin of the Xist Gene from a Protein-Coding Gene and a Set of Transposable Elements
Source: PLoS One. 2008 Jun 25;3(6):e2521. doi: 10.1371/journal.pone.0002521 (PMC2430539; doi:10.1371/journal.pone.0002521)
Supplement: Table S2 — (0.04 MB DOC) [file pone.0002521.s008.doc]

**Table S2. Repeat composition of the *Xist* gene in *Canis familiaris* (C.f.), *Bos taurus* (B.t.), *Homo sapiens* (H.s.), *Pan troglodytes* (P.t.), *Rattus norvegicus* (R.n.); *Mus musculus* (M.m.); *Microtus rossiaemeridionalis* (M.r.), and eutherian consensus (con.) *Xist* gene.**

|  | Various type of repeats in Xist in percent | | | | | | | | |
| --- | --- | --- | --- | --- | --- | --- | --- | --- | --- |
| **Species** | **Region analyzed**  **length bp** | GC  level in % | SINEs | LINEs | LTR  Ele-ments | **DNA**  **Ele-ments** | Simple repeats | Low comp-lexity | Total  intersp.  repeats |
| **C.f.** | **38592** | 38.9 | 5.77 | 8.55 | - | **0.35** | 1.31 | - | 15.97 |
| **B.t.** | **35934** | 40.3 | 5.96 | 7.43 | 0.20 | **0.74** | 0.83 | - | 15.26 |
| **H.s.** | **33063** | 39.8 | 6.37 | 4.55 | - | 0.52 | **0.68** | 0.28 | 12.40 |
| **P.t.** | **32708** | 39.6 | 6.40 | 4.85 | - | 0.53 | **0.36** | - | 12.13 |
| **R.n.** | **23898** | 42.4 | 6.95 | - | - |  | **0.95** | - | 7.90 |
| **M.m.** | **23786** | 41.3 | 6.95 | - | - | 0.30 | **2.18** | 0.70 | 10.12 |
| **M.r.** | **22163** | 42.2 | 7.64 | - | - | 0.28 | **1.76** | 1.31 | 10.99 |
| **con.** | **30986** | 37.7 | 1.64 | 0.46 | - | 0.51 | **0.84** | 0.93 | 4.39 |

**Note. Analyzed region in each species includes 500bp of 5’-flank, *Xist* gene and 500bp of 3’-flank.**
