# Supplementary material for: A Dual Origin of the Xist Gene from a Protein-Coding Gene and a Set of Transposable Elements
Source: PLoS One. 2008 Jun 25;3(6):e2521. doi: 10.1371/journal.pone.0002521 (PMC2430539; doi:10.1371/journal.pone.0002521)
Supplement: Table S3 — (0.03 MB DOC) [file pone.0002521.s009.doc]

**Table S3. Homology (%) of P-min promoter sequence of *Xist* gene in members of nine eutherian species.**

|  | CHIMP | DOG | HUMAN | MOUSE | RAT | VOLE | ANCESTOR |
| --- | --- | --- | --- | --- | --- | --- | --- |
| CATTLE | 83 | 92 | 84 | 69 | 70 | 68 | 91 |
| CHIMP |  | 88 | 98 | 76 | 78 | 72 | 97 |
| DOG |  |  | 89 | 72 | 74 | 71 | 95 |
| HUMAN |  |  |  | 74 | 78 | 71 | 98 |
| MOUSE |  |  |  |  | 90 | 76 | 80 |
| RAT |  |  |  |  |  | 75 | 83 |
| VOLE |  |  |  |  |  |  | 77 |
