# Supplementary material for: A Dual Origin of the Xist Gene from a Protein-Coding Gene and a Set of Transposable Elements
Source: PLoS One. 2008 Jun 25;3(6):e2521. doi: 10.1371/journal.pone.0002521 (PMC2430539; doi:10.1371/journal.pone.0002521)
Supplement: Table S4 — (0.03 MB DOC) [file pone.0002521.s010.doc]

**Table S4. Branch lengths estimated using different approaches.**

| Models of substitutions | B1_N_ | B2_N_ | B_S_ |
| --- | --- | --- | --- |
| Maximum likelihood | 0.486 | 0.196 | 0.232 |
| Maximum likelihood, CpGs removed | 0.456 | 0.178 | 0.247 |
| Modified Nei-Gojobori method, p-distance | 0.264 | 0.114 | 0.158 |
| Modified Nei-Gojobori method, JC-correction, CpGs removed | 0.577 | 0.261 | 0.440 |

B1_N_, B2_N_ and B_S_ are the branches of phylogenetic trees shown in the Figure 3.
