# Supplementary material for: A Dual Origin of the Xist Gene from a Protein-Coding Gene and a Set of Transposable Elements
Source: PLoS One. 2008 Jun 25;3(6):e2521. doi: 10.1371/journal.pone.0002521 (PMC2430539; doi:10.1371/journal.pone.0002521)
Supplement: Table S5 — (0.06 MB DOC) [file pone.0002521.s011.doc]

**Table S5. A list of all gene names, their locations, and symbols used in the paper.**

| Gene symbol | Gene name | H.sapiens | M.domestica | G.gallus | X.trpicalis | D.rerio | O.latypus | T. rubripes | T.nigroviridis |
| --- | --- | --- | --- | --- | --- | --- | --- | --- | --- |
| Xist | the X-inactive specific transcript | Chr. X |  |  |  |  |  |  |  |
| Tsix | Xist antisense RNA | Chr. X |  |  |  |  |  |  |  |
| Lnx3 | ligand of numb-protein X 3 |  | Chr. X | Chr. 4 | Scaffold 10 | Chr. 14 | Chr. 10 | Scaffold 132 | Scaffold 22736 |
| Tsx | testis specific X-linked gene | Chr. X |  |  |  |  |  |  |  |
| Enox(Jpx) | Expressed neighbour of Xist | Chr. X |  |  |  |  |  |  |  |
| Ftx | Mus musculus Ftx noncoding RNA | Chr. X |  |  |  |  |  |  |  |
| Cnbp2 | cellular nucleic acid binding protein 2 | Chr. X |  |  |  |  |  |  |  |
| Fip1l2 | Polyadenylation factor I complex (Saccharomyces cerevisiae), like 2 |  |  | Chr. 4 |  |  |  |  |  |
| Rasl11c | RAS-like, family 11, member C |  | Chr. X | Chr. 4 |  |  |  |  |  |
| Uspl2 | ubiquitin specific peptidase |  |  | Chr. 4 |  |  |  |  |  |
| Wasf3(Wave4) | Wiskott-Aldrich syndrome protein family member 3 |  |  | Chr. 4 |  |  |  |  |  |
| Cdx4 | caudal type homeo box transcription factor 4 | Chr. X | Chr. X | Chr. 4 | Scaffold 10 | Chr. 14 | Chr. 10 | Scaffold 132 |  |
| Chic1 | cysteine-rich hydrophobic domain 1 | Chr. X | Chr. X | Chr. 4 | Scaffold 10 | Chr. 14 | Chr. 10 | Scaffold 132 | Scaffold 22736 |
| Slc16a2 | solute carrier family 16 | Chr. X | Chr. X | Chr. 4 |  |  |  |  |  |
| Rnf12 | ring finger protein 12 | Chr. X | Chr. X | Chr. 4 | Scaffold 10 | Chr. 14 |  |  |  |
| Kiaa2022 | hypothetical protein 2022 | Chr. X | Chr. X | Chr. 4 |  |  |  |  |  |
| Abcb7 | ATP-binding cassette, sub-family B, member 7 | Chr. X | Chr. X | Chr. 4 | Scaffold 10 | Chr. 14 |  |  |  |
| Kdr | kinase insert domain receptor |  |  |  | Scaffold 10 | Chr. 14 |  |  |  |
| Slc25a4 | solute carrier family 25. |  |  |  |  |  | Chr. 10 | Scaffold 132 |  |
